# Establishing a common metric for patient-reported outcomes in cancer patients: linking patient reported outcomes measurement information system (PROMIS), numerical rating scale, and patient-reported outcomes version of the common terminology criteria for adverse events (PRO-CTCAE)

**DOI:** 10.1186/s41687-020-00271-0

**Published:** 2020-12-10

**Authors:** Minji K. Lee, Benjamin D. Schalet, David Cella, Kathleen J. Yost, Amylou C. Dueck, Paul J. Novotny, Jeff A. Sloan

**Affiliations:** 1 Robert D. and Patricia E. Kern Center for the Science of Health Care Delivery, Mayo Clinic, 200 First St SW, Rochester, MN 55906 USA; 2 Department of Medical Social Sciences, Northwestern University Feinberg School of Medicine, 625 Michigan Ave, 27th Floor, Chical, IL 60611 USA; 3 Department of Health Sciences Research, Mayo Clinic, 200 First St SW, Rochester, MN 55905 USA; 4 Department of Health Sciences Research, Mayo Clinic, 13400 E. Shea Blvd, Scottsdale, AZ 85259 USA

**Keywords:** Linking, PROMIS, PRO-CTCAE, NRS

## Abstract

**Background:**

Researchers and clinicians studying symptoms experienced by people with cancer must choose from various scales. It would be useful to know how the scores on one measure translate to another.

**Methods:**

Using item response theory (IRT) with the single-group design, in which the same sample answers all measures, we produced crosswalk tables linking five 0–10 numeric rating scale (NRS) and 15 items from Patient-Reported Outcomes version of the Common Terminology Criteria for Adverse Events (PRO-CTCAE, scored on a 1–5 scale) to the T-Score metric of six different scales from the NIH Patient reported Outcomes Measurement Information System (PROMIS®). The constructs, for which we conducted linking, include emotional distress-anxiety, emotional distress-depression, fatigue, sleep disturbance, pain intensity, and pain interference. We tested the IRT linking assumption of construct similarity between measures by comparing item content and testing unidimensionality of item sets comprising each construct. We also investigated the correlation of the measures to be linked and, by inspecting standardized mean differences, whether the linkage is invariant across age and gender subgroups. For measures that satisfied the assumptions, we conducted linking.

**Results:**

In general, an NRS score of 0 corresponded to about 38.2 on the PROMIS T-Score scale (mean = 50; SD = 10); whereas an NRS score of 10 corresponded to a PROMIS T-Score of approximately 72.7. Similarly, the lowest/best score of 1 on PRO-CTCAE corresponded to 39.8 on *T-score* scale and the highest/worst score of 5 corresponded to 72.0.

**Conclusion:**

We produced robust linking between single item symptom measures and PROMIS short forms.

**Supplementary Information:**

The online version contains supplementary material available at 10.1186/s41687-020-00271-0.

Patient-reported outcome measures (PROMs) are tools for directly eliciting patient experience; their use has become the standard in clinical trials for assessing symptoms and health-related quality of life (HRQOL) [1, 2]. Single-item measures have been used for the simplicity of administration, reduction in respondent burden, and ease of interpretation [3, 4]. Validity of single-item numerical rating scales (NRS) has been demonstrated and in some settings, they are an efficient alternative to longer assessments [1, 4, 5].

Commonly used measures in oncology include PRO-CTCAE (PRO version of the Common Terminology Criteria for Adverse Events) and PROMIS® (PRO Measurement Information System) [1]. The PRO-CTCAE was designed to assess side-effects related to treatment toxicity or tolerability. PROMIS provides short forms for a number of selected symptoms and HRQOL, which vary in length to meet the needs of researchers, balancing a tradeoff between precision and respondent burden.

Given this heterogeneity of PROMs, it would be useful to know how the scores on single-item measures such as NRS or PRO-CTCAE map onto longer, calibrated PROMIS scales measuring the same construct. These maps, or cross-walk tables, would allow researchers and clinicians to more accurately compare results across studies that use different PROMs, and allow for a common reporting metric in comparative effectiveness research or meta-analyses. Once multiple instruments are linked on cross-walk tables, clinicians and investigators can determine if clinical cutoff scores on different instruments converge or diverge based on a common metric [6].

Previous studies have linked legacy measures to the PROMIS T-score metric for depression [6, 7], anxiety [8], pain interference [9], physical function [10], and fatigue [11]. The current study is the first to link NRS and PRO-CTCAE single-item measures to their associated PROMIS short form measures. This allows placing all measures on the same (PROMIS) metric. We present the cross-walk results on the following domains: fatigue, pain intensity, pain interference, sleep disturbance, anxiety, and depression.

## Methods

### Sample

Adult cancer patients were recruited from five cancer centers: University of North Carolina, Memorial Sloan-Kettering Cancer Center, Northwestern University, MD Anderson Cancer Center, and Mayo Clinic in Rochester, Minnesota. Patients were eligible for the study if they had a diagnosis of cancer, were currently receiving anti-cancer treatment or would be initiating active anti-cancer treatment within the next 7 days, or underwent surgery for cancer treatment in the past 14 days. Patients treated with only hormonal therapy and patients with clinically significant cognitive impairment were excluded. The study was reviewed by the IRB of each of the participating sites, and all patients provided consent to enter the study. Patients were randomized to three modes of administration for the baseline assessment: paper, IVRS, and web. All follow-up assessments at 6 weeks were administered by mail. The current study utilizes the baseline data only.

### Measures

#### Pro-CTCAE

PRO-CTCAE is a patient version of the existing clinician-reported adverse event items for use in cancer clinical trials. The intent of PRO-CTCAE is to improve the accuracy and precision of adverse symptom assessment in cancer trials, and to bring the CTCAE into harmony with other areas of clinical research, in which the gold standard for symptom evaluation is patient self-report. The PRO-CTCAE consists of five types of items (presence/absence, amount, frequency, severity, and interference with usual or daily activities). For items asking the frequency of symptoms, the response options are (a) never, (b) rarely, (c) occasionally, (d) frequently, and (e) almost constantly. For severity items, response options are (a) none, (b) mild, (c) moderate, (d) severe, and (e) very severe. The response options for items asking interference with daily activities include (a) not at all, (b) a little bit, (c) somewhat, (d) quite a bit, and (e) very much.

#### NRS

The NRS items have 11 response options from 0 to 10, but the interpretation of high scores varies by domain. For pain, fatigue, anxiety and depression, an NRS score of 0 indicates the patient does not experience the symptom and 10 indicates the symptom is as bad as imaginable. Conversely, for overall QOL, emotional/mental/physical well-being, social activity, and sleep quality an NRS score of 0 as bad as it can be and 10 indicates the best it can be. The NRS item for sleep quality was reverse-coded to represent sleep disturbance with similar interpretation to the other symptoms (pain, fatigue, etc).

#### PROMIS

We administered six version 1.0 short forms derived from PROMIS item banks: Anxiety 8a, Depression 8a, Fatigue 7a with two additional items from Fatigue 8a (i.e., FATIMP3, FATIMP16), Sleep Disturbance 8a, Pain Intensity 3a, Pain Interference 8a excluding one item (8a-1) as it was redundant with a PRO-CTCAE item (i.e., In the past 7 days, how much did pain INTERFERE with your usual or daily activities?), and Physical Function 10a. We used the version 2.0 short form 8a for Ability to Participate in Social Roles and Activities. The PROMIS measures are scored on a *T* score metric in which 50 is the mean of a general US adult reference population and 10 is the standard deviation (SD) of that reference population.

### Linking design

Following the methods of previously linking studies with PROMIS measures [6–11], we used the single-group design, in which the same sample answers all three measures (PROMIS; NRS; PRO-CTCAE). This is the strongest of the linking methodologies [12]. We used two IRT-based linking methods: Fixed-parameter calibration, and concurrent calibration followed by transformation with linking constants [13, 14]. When the two approaches provide the same result, a robust linking relationship between instruments can be obtained.

#### Fixed-parameter calibration

In the fixed-parameter calibration, the item parameters of the anchor measure (PROMIS) were fixed at their previously established calibration [6, 15], while the item parameters of the target measures (NRS or PRO-CTCAE) were freely estimated (subject to the metric defined by the anchor measure) in a single run for each domain. For example, in anxiety domain, there were 8 PROMIS items, 1 NRS, and 3 PRO-CTCAE items. These single-item measures were calibrated in a single run. Afterwards, each single item measure was anchored to the metric defined by the PROMIS item parameters. This calibration yielded item parameters for the legacy measure that were on the PROMIS metric.

#### Concurrent calibration with linking constants

The second IRT-based method we applied was concurrent calibration followed by the computation of transformation constants. With concurrent calibration, all items of the anchor and target measures are freely estimated in a single calibration. This produces a common metric and avoids imposing the constraints inherent in the fixed-parameter calibration (e.g., differences in population). However, the item statistics (calibrations) are arbitrary, or not linked to the original anchor item calibrations. To address this, linking constants are derived from the difference between these new “free” PROMIS calibrations and the previously established PROMIS calibrations. These constants are multiplicative and additive constants from the two sets of parameters so that their test characteristic curves (TCCs) become as similar as possible [14]. These constants can then be applied to the free calibrations of the target measures, thereby putting their parameters on the common metric. A test characteristic curve method by Stocking and Lord [14] was used to obtain the linking constants with an R package, lordif [16]. We ran all calibrations using flexMIRT® [17].

### Tests of linking assumptions

The first linking assumption is construct similarity between measures [12, 18]. When two measures are developed using different test specifications but measure similar constructs, we can produce concordance table that transforms scores from one to another. To test the similarity of constructs, we used several methods. First, we evaluated the degree of conceptual interchangeability by inspecting item content across measures. Second, since our planned IRT calibrations require that the combined item set is unidimensional, we conducted the confirmatory factor analyses (CFA) treating the items as ordinal and using WLSMV estimator with lavaan R package [19]. Using commonly used benchmark values [20], model fit was evaluated based on standard fit indices including the Comparative Fit Index (CFI ≥ 0.95 very good fit) and the Standardized Root Mean Square Error Residual (SRMR ≤0.08). We also estimated the proportion of total variance attributable to a general factor (i.e., coefficient omega, ω_h_) [21, 22] using the psych package [23] in R. This method estimates ω_h_ from the general factor loadings derived from principal axes factor analysis and a Schmid-Leiman transformation [24]. The default was to extract 3 group factors, and for two domains, two subfactors had more desirable solutions. Values of .70 or higher for ω_h_ suggest that the item set is sufficiently unidimensional for most analytic procedures that assume unidimensionality [25].

A second linking assumption is that the scores of the two measures to be linked are highly correlated [18]. We calculated correlation coefficients between the raw scores of the measures to be linked. We evaluated a third linking assumption (i.e., linkage is invariant across important subgroups) by computing mean differences between important subpopulations [18]. We chose two types of subgroups based on gender and age (i.e., men and women; ages ≥60 and ages < 60). To compute the standardized mean difference (*smd*) between males and females, the difference between female and male means was divided by the total group pooled standard deviation. If the smd values of PROMIS and the measures to be linked are similar (≤ 0.10), then we can assume linkage is likely invariant between subgroups. A difference in SMDs greater than 0.11 suggests a need for sub-population-specific cross-walks [12, 18].

## Results

### Sample

As displayed in Table 1, the mean age of 1859 patients was 56 years. There were more women (61%) than men (39%) in the sample. About 74% were Caucasians, 22% Blacks, 3% Asians, 0.3% American Indians or Alaska Natives. About 6% were Hispanics. Breast cancer, lymphoma/myeloma, colorectal cancer, head/neck/gastroesophageal cancer, and lung cancer made up 71.2% of the patients. There were 12% of the patients who were in stage I cancer, 21% in stage II, 30% in stage III, and 37% in stage IV. There were 6% who had education less than high school, 23% high school or GED, 30% some college, and 41% college graduate or more.

**Table 1:** Demographic Information (*N* = 1859)

**Assessment Condition**	
IVRS	602 (32.4%)
Paper	654 (35.2%)
Web	603 (32.4%)
**Treatment Site**	
MD Anderson	354 (19.0%)
Mayo Clinic	858 (46.2%)
Memorial-Sloan Kettering	149 (8.0%)
Northwestern University	434 (23.3%)
University of North Carolina	64 (3.4%)
**Age**	
Mean (SD)	56.4 (12.5)
Q1, Median, Q3	49.0, 58.0, 65.0
Range	(18.0–89.0)
**Gender**	
F	1131 (61.0%)
M	722 (39.0%)
Missing	6
**Race: On Study Form**	
White	1367 (73.8%)
Black or African American	407 (22.0%)
Asian	54 (2.9%)
American Indian or Alaska Native	5 (0.3%)
Not reported: patient refused or not available	10 (0.5%)
Unknown: Patient unsure	10 (0.5%)
Missing	6
**Ethnicity: On Study Form**	
Hispanic or Latino	106 (5.7%)
Not Hispanic or Latino	1729 (93.3%)
Not reported: Patient refused or data not available	12 (0.6%)
Unknown: Patient is unsure of their ethnicity	6 (0.3%)
Missing	6
**Disease**	
Breast	462 (25.9%)
Lymphoma/myeloma	370 (20.8%)
Prostate/bladder	21 (1.2%)
Lung	136 (7.6%)
Colorectal	177 (9.9%)
Head/neck/gastroesophageal	158 (8.9%)
Other	457 (25.7%)
Missing	78
**PS on Checklist**	
0	853 (46.0%)
1	838 (45.2%)
2	139 (7.5%)
3	22 (1.2%)
4	1 (0.1%)
Missing	6
**Disease Stage**	
I	207 (11.8%)
II	375 (21.4%)
III	518 (29.5%)
IV	654 (37.3%)
Missing	105
**Education Level: On Study Form**	
Less Than High School	104 (5.9%)
High School or GED	413 (23.3%)
Some College	524 (29.5%)
College Graduate or More	735 (41.4%)
Missing	83

### Assumptions

#### Construct similarity

Content of the items from three kinds of measures representing each construct was similar. The PROMIS Anxiety 8a consists of statements on the frequency of feeling nervous, anxious, tense, and feeling fearful, and the NRS asks the severity and PRO-CTCAE asks the severity, frequency, interference of anxiety. In addition, the content in single-item measures was fully represented in PROMIS Depression 8a such as feeling depressed, unhappy, or nothing could cheer one up, but the PROMIS focused on the frequency of these feelings while the single-item measures asked these feelings in terms of frequency, severity and interference. PROMIS Depression 8a had other content not represented in NRS or PRO-CTCAE such as feeling worthless, helpless, hopeless, feeling like a failure, or having nothing to look forward to. For fatigue, the single-item measures directly asked the level of fatigue and its interference with activities, while PROMIS items mostly addressed the construct without mentioning “fatigue”. For example, PROMIS asked the frequency of feeling tired, experiencing extreme exhaustion, running out of energy, or feeling too tired to think clearly or take a bath. There was one PROMIS item that asked how often fatigue interferes with work. Regarding sleep, both NRS and PROMIS had an item addressing average sleep quality. Many items in Sleep Disturbance 8a asked about sleep difficulty in a variety of ways, such as the degree to which sleep was refreshing, restless, or satisfying, and how hard it was to fall asleep. The related PRO-CTCAE items asked the severity of insomnia at its worst and its interference with activities.

The content of both NRS and PRO-CTCAE items addressing pain intensity was fully represented in Pain Intensity 3a which asked how intense one’s pain was at its worst, average pain intensity, and the level of pain right now. There was no NRS item addressing pain interference. A PRO-CTCAE item asked the degree to which pain interfered with activities in general, whereas Pain Interference 8a-1 asked interference with different aspects of activities such as working around the home, social activities, family life, or household chores. Similarly, the PROMIS short forms for social and physical function covered various aspects of the functions, while the NRS counterparts asked for global ratings of social activity and physical well-being.

For the item sets combining PROMIS, NRS, and PRO-CTCAE, CFA fit statistics were excellent, depending on the fit statistics referenced. For anxiety domain, fit values were CFI = 0.99, TLI = 0.987, SRMR = 0.045; for depression, fit values were CFI = 0.993, TLI = 0.991, SRMR = 0.042; for fatigue, CFI = 0.993, TLI = 0.992, SRMR = 0.036; for fatigue, CFI = 0.993, TLI = 0.992, SRMR = 0.036; for sleep disturbance, CFI = 0.987, TLI = 0.984, SRMR = 0.066; for pain intensity, CFI = 1, TLI = 1, SRMR = 0.016; for pain interference, CFI = 1, TLI = 0.999, SRMR = 0.012; and for ability to participate in social roles and activities, CFI = 0.998, TLI = 0.998, SRMR =0.025. The results suggest essential unidimensional data-model fit. The coefficient omega (ω_h_) values were .88 for anxiety, .89 for depression, .88 for fatigue, .80 for sleep disturbance, .91 for pain intensity, .96 for pain interference, .93 for social function, .80 for physical function, and .75 for global mental health, supporting the presence of a dominant general factor for each combination of instruments.

#### Correlations between measures to be linked

The Pearson correlation between PROMIS and NRS, or PROMIS and PRO-CTCAE items ranged from .70 to .77 for anxiety; .72 to .79 for depression; .76 to .82 for fatigue; .79 to .85 for sleep disturbance; .87 to .89 for pain intensity; and .88 for pain interference (Table 2). The correlations for the following domains were deemed too low to support linking: .65 for social function; .57 to .64 for global mental health; and .53 for physical function. Choi et al. [6] suggested a lower bound of correlation of .75 for scores to be linked.

**Table 2:** List of Domains and Measures Considered for the Linking

Domain	Single-Item Assessment	PROMIS short forms	***r***^a^	Linked	Reason if excluded from linking
Anxiety	NRS: During the past week, including today, how would you describe your level of anxiety, on the average?	Emotional Distress-Anxiety 8a	.70	Yes	
	PRO-CTCAE: In the past 7 days, what was the severity of your anxiety at its worst?		.75	Yes	
	PRO-CTCAE: In the past 7 days, how often did you feel anxiety?		.77	No	Difference in smd’s by age > 0.1^b^
	PRO-CTCAE: In the past 7 days, how much did anxiety interfere with your usual or daily activities?		.74	No	Difference in smd’s by age > 0.1
Depression	NRS: During the past week, including today, how would you describe your level of depression, on the average?	Emotional Distress-Depression 8a	.78	Yes	
	PRO-CTCAE: In the past 7 days, how often did you feel that nothing could cheer you up?		.78	Yes	
	PRO-CTCAE: In the past 7 days, what was the severity of your feelings that nothing could cheer you up at the worst?		.79	Yes	
	PRO-CTCAE: In the past 7 days, how much did feeling that nothing could cheer you up interfere with your usual or daily activities?		.75	Yes	
	PRO-CTCAE: In the past 7 days, how often did you have sad or unhappy feelings?		.74	Yes	
	PRO-CTCAE: In the past 7 days, what was the severity of your sad/unhappy feelings at their worst?		.72	Yes	
	PRO-CTCAE: In the past 7 days, how much did sad or unhappy feelings interfere you’re your usual or daily activities?		.76	Yes	
Fatigue	NRS: During the past week, including today, how would you describe your level of fatigue on average?	Fatigue custom form (7a + 2)	.76	Yes	
	PRO-CTCAE: In the past 7 days, what was severity of your fatigue, tiredness, or lack of energy at its worst?		.76	Yes	
	PRO-CTCAE: In the past 7 days, how much did fatigue, tiredness, or lack of energy interfere with your usual or daily activities?		.82	Yes	
Sleep	NRS: During the past week, including today, how would you describe the quality of your sleep on average?	Sleep Disturbance 8a	.85	Yes	
	PRO-CTCAE: In the past 7 days, what was the severity of your insomnia including difficulty falling asleep, staying asleep, or waking up early at its worst?		.84	Yes	
	PRO-CTCAE: In the past 7 days, how much did insomnia including difficulty falling asleep, staying asleep, or waking up early interfere with your usual or daily activities?		.79	Yes	
Pain Intensity	NRS: During the past week, including today, how would you describe the severity of your pain on average?	Pain Intensity 3a	.87	Yes	
	PRO-CTCAE: In the past 7 days, what was severity of your pain at its worst?		.89	Yes	
Pain Interference	PRO-CTCAE: In the past 7 days, how much did pain interfere with your usual or daily activities?	Pain Interference custom form (8a-1)	.88	Yes	
Social function	NRS: During the past week, including today, how would you describe your level of social activity?		.65	No	Low correlation
Physical function	NRS: During the past week, including today, how would you describe your overall physical well being?		.53	No	Low correlation
Global mental health	NRS: During the past 7 days, including today, how would you describe your overall quality of life?		.57	No	Low correlation
	NRS: During the past week, including today, how would you describe your overall emotional well-being?		.64	No	Low correlation
	NRS: During the past week, including today, how would you describe your overall mental (intellectual) well-being?		.59	No	Low correlation

^a^*r* denotes correlation

^b^Difference in *smd* (i.e., standardized mean difference) in two age groups (ages ≥60 and ages < 60) between the PROMIS short form and the single-item assessment was greater than 0.1

#### Invariant linkage between subgroups

**Fig. 1:**
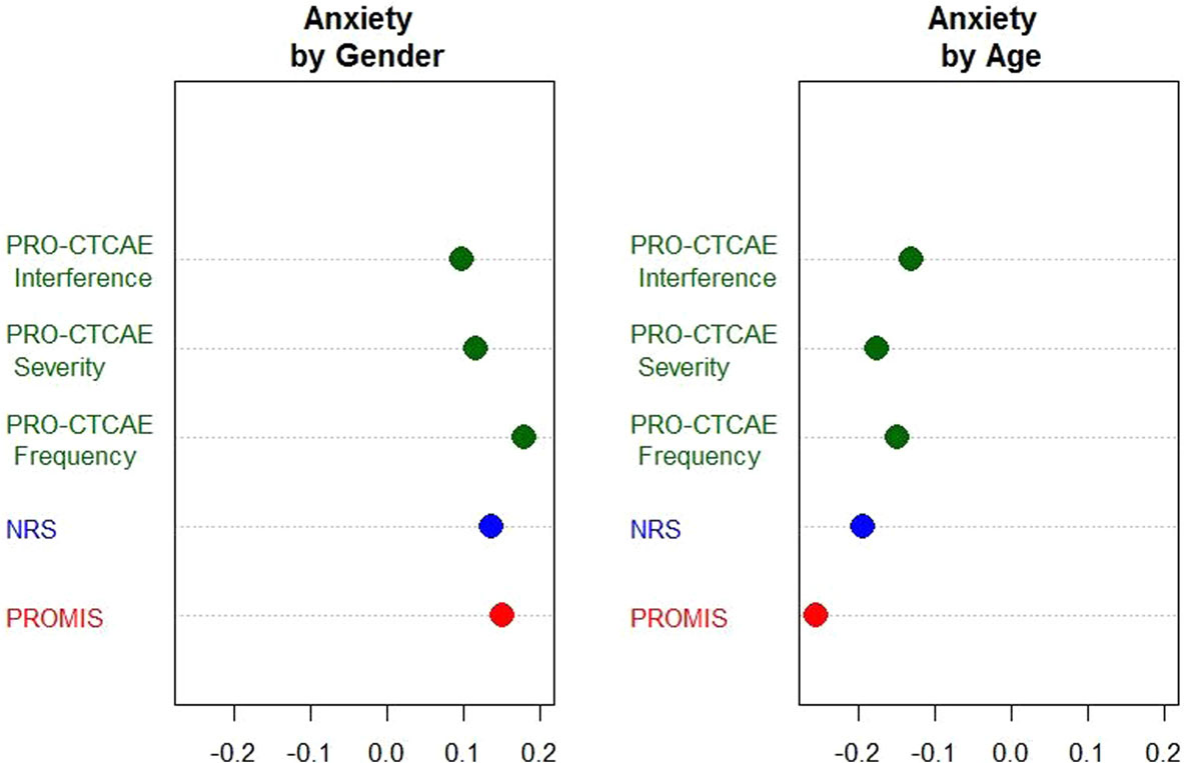
Standardized Mean Differences by Gender and Age (< 60 vs ≥ 60). Average Female subtracted by male scores, and average older (≥ 60) subtracted by younger (< 60) scores are presented for anxiety scales

As shown in Fig. 1 (anxiety domain as an example) Appendix A (for all scales), the smd’s by gender between PROMIS and other measures were similar (≤ 0.1 difference). Those by age were more variable for anxiety domain: The smd’s of PRO-CTCAE frequency (− 0.15) and interference (− 0.13) items were at least 0.11 point away [18] from the smd of PROMIS by age (− 0.26). This suggests that the linking relationship of PRO-CTCAE frequency and interference items to PROMIS anxiety scales may not be the same for the older and the younger patients. For five other domains, the smd’s by gender or age between PROMIS and other measures were sufficiently close. On the basis of the findings above, the final scales to be linked were determined (Table 2).

### Linkage results

**Fig. 2:**
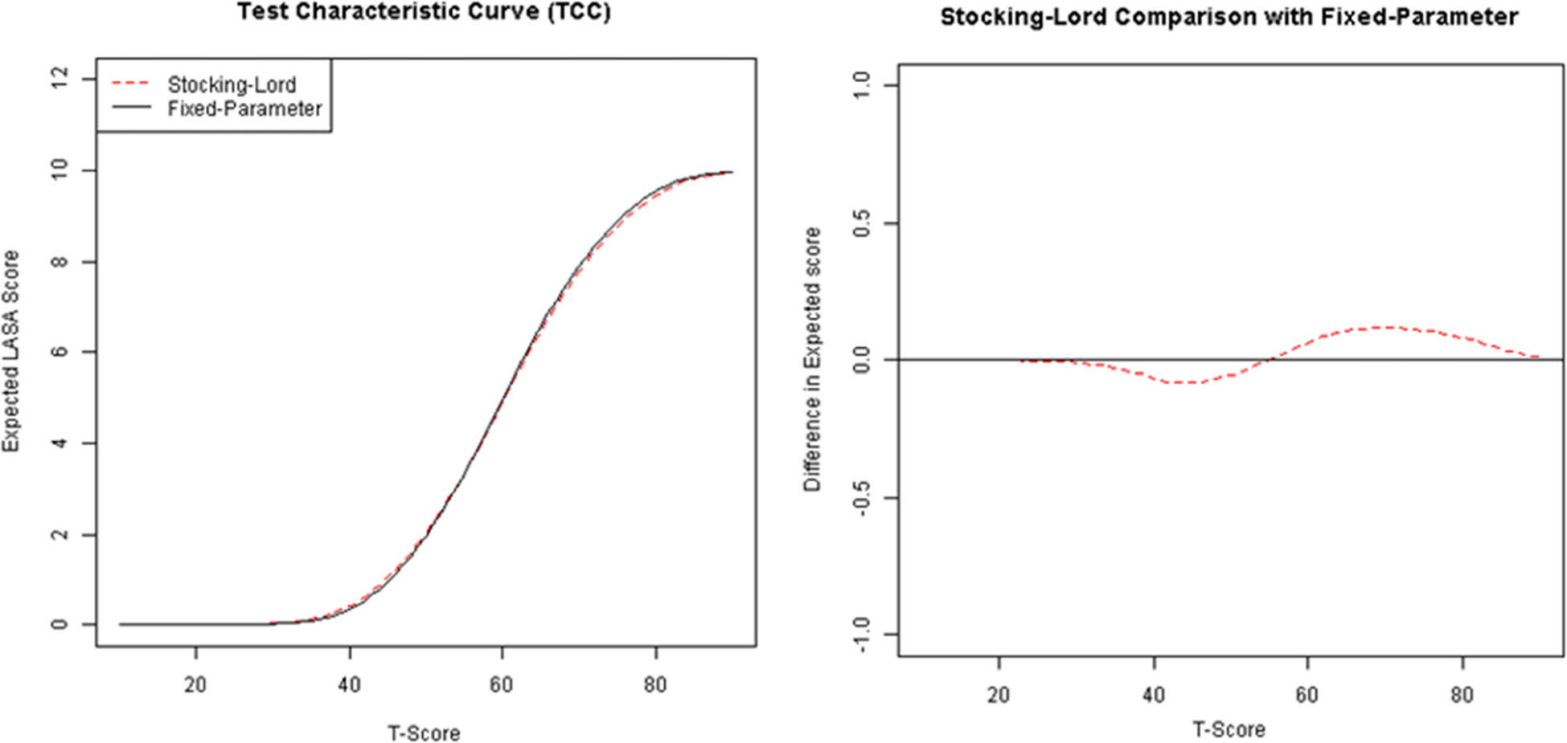
Comparison of Test Characteristic Curves in NRS Anxiety scale and the Difference in Raw Score Values across the Scale between Concurrent Calibration with Linking Constants and Fixed Calibration

Discrimination and location parameters on the PROMIS metric were estimated for the PRO-CTCAE and NRS items. Based on these parameters, we plotted the test characteristic curves (TCCs), showing the score values of the non-PROMIS items on the y-axis against the corresponding PROMIS-Tscore on the x-axis (anxiety NRS as an example in Fig. 2; all scales in Appendix B). Furthermore, we plotted the differences of the fixed calibration method vs the concurrent calibrations using linking constants (Fig. 2 and Appendix B). For each comparison between the TCCs, the expected raw score value differed by less than 1 point across thetas ranging from − 4 to 4. For all domains except pain intensity, the expected raw score values differed by less than |0.5| point across thetas. For pain intensity, in a higher T-score range of about 60–80, the difference in NRS score was larger than |0.5|. Because of the close similarity of the two IRT solutions on most of the domains and because the concurrent calibration using linking constants makes fewer assumptions about the population difference of the current sample and the original PROMIS calibration sample, we report only the results of the concurrent calibration followed by linking constants. The fixed and freely estimated item parameters of the PROMIS anchor items are plotted along with the identity line in Appendix C, which shows how the two calibrations compare to each other.

Cross-walk tables based on concurrent calibration followed by transformation with Stocking-Lord linking constants are provided in Tables 3, 4 and 5. Table 6 shows the item parameters for NRS and PRO-CTCAE items from concurrent calibrations with linking constants. We mapped the raw scores on NRS or PRO-CTCAE to their corresponding PROMIS *T* scores based on the conversion tables constructed with Lord & Wingersky method [26].

**Table 3:** Cross-walk Table for NRS items using Concurrent Calibration followed by Stocking-Lord Linking Constants

	Fatigue		Pain Intensity		Sleep Disturbance^b^		Anxiety		Depression	
	T-Score (SE)	N^a^	T-Score (SE)	N	T-Score (SE)	N	T-Score (SE)	N	T-Score (SE)	N
0	37.6 (6.3)	197	37.8 (6.4)	579	35.0 (6.9)	174	39.9 (6.8)	500	41.5 (6.7)	860
1	44.7 (4.8)	247	45.7 (5)	258	41.0 (5.8)	228	47.4 (5.1)	300	49.9 (4.3)	295
2	48.2 (4.6)	210	50.4 (4.8)	223	45.5 (5.6)	316	50.9 (5.0)	245	52.9 (4.3)	185
3	50.9 (4.6)	226	54.1 (4.8)	185	49.4 (5.4)	276	53.6 (5.0)	173	55.4 (4.3)	140
4	53.1 (4.6)	146	56.9 (4.8)	105	52.3 (5.4)	193	55.6 (5.1)	117	57.4 (4.3)	79
5	55.3 (4.7)	235	59.3 (4.9)	134	55.0 (5.5)	224	57.4 (5.1)	135	59.4 (4.4)	80
6	57.8 (4.7)	166	62.2 (5.1)	109	57.6 (5.6)	127	59.4 (5.2)	104	61.3 (4.4)	38
7	60.5 (4.9)	188	64.9 (5.3)	83	60.3 (5.8)	122	61.6 (5.4)	91	63.3 (4.6)	50
8	64.3 (5.2)	112	68.1 (5.7)	66	63.6 (6.2)	76	64.1 (5.6)	66	66.2 (4.8)	26
9	68.0 (5.5)	29	71.4 (6.1)	23	66.7 (6.5)	23	67.4 (6.1)	35	69.2 (5.0)	11
10	72.2 (6.4)	17	74.9 (6.8)	17	70.1 (7.4)	20	71.9 (7.0)	10	73.5 (5.9)	8

^a^N denotes the sample size for each score on the NRS item

^b^The NRS item asked sleep quality rather than sleep disturbance, so was reverse-coded

**Table 4:** Cross-walk Table for the PRO-CTCAE items for fatigue, pain intensity, pain interference, and sleep disturbance

	Fatigue-Severity of fatigue at the worst		Fatigue-Interference with activities		Pain Intensity-Severity of pain at its worst		Pain Interference-Interference with activities		Sleep-Severity of insomnia at its worst		Sleep-Interference with activities	
	T-Score (SE)	N^a^	T-Score (SE)	N	T-Score (SE)	N	T-Score (SE)	N	T-Score (SE)	N	T-Score (SE)	N
1	37.8 (6.1)	222	40.0 (6.1)	402	36.2 (5.7)	551	43.3 (6.8)	844	39.7 (6.5)	550	42.1 (7.1)	762
2	47.7 (4.8)	648	50.0 (4.0)	607	47.5 (5.0)	541	54.2 (4.0)	440	48.7 (4.8)	511	51.4 (5.4)	518
3	55.8 (4.7)	628	56.7 (3.8)	469	57.1 (4.5)	419	59.8 (3.9)	290	55.7 (5.0)	517	57.5 (5.5)	342
4	63.0 (4.7)	224	63.1 (4.1)	244	65.5 (4.6)	216	65.2 (4.1)	156	63.2 (5.2)	170	63.5 (6.0)	131
5	70.2 (5.7)	52	71.1 (4.9)	49	74.0 (5.2)	55	71.9 (5.1)	53	70.6 (6.0)	34	70.0 (6.9)	25

^a^N denotes the sample size for each score on the PRO-CTCAE item

**Table 5:** Cross-walk Table for the PRO-CTCAE items for anxiety and depression

	Anxiety-Severity of anxiety at the worst		Depression Frequency-Feeling nothing could cheer you up		Depression Severity-Feeling nothing could cheer you up		Depression Interference-Nothing could cheer you up		Depression Frequency- Sad/unhappy feelings		Depression Severity-Sad/unhappy feelings		Depression Interference-Sad/unhappy feelings	
	T-Score (SE)	N^a^	T-Score (SE)	N	T-Score (SE)	N	T-Score (SE)	N	T-Score (SE)	N	T-Score (SE)	N	T-Score (SE)	N
1	40.7 (6.6)	570	43.0 (6.9)	1065	43.6 (6.9)	1160	44.4 (7.2)	1248	38.4 (6.1)	509	39.7 (6.3)	629	43.5 (7.1)	1128
2	51.0 (4.9)	563	53.5 (4.3)	436	54.6 (4.0)	370	55.8 (4.3)	342	48.4 (4.8)	729	50.2 (5.0)	772	54.3 (4.5)	422
3	58.6 (4.9)	460	59.6 (4.5)	203	60.8 (4.2)	180	61.5 (4.4)	122	56.3 (4.6)	397	58.5 (4.8)	299	60.3 (4.5)	154
4	65.5 (5.1)	162	65.4 (4.6)	53	67.2 (4.2)	42	66.7 (4.6)	42	63.7 (4.9)	116	65.0 (4.7)	50	65.7 (4.8)	51
5	72.4 (5.9)	21	71.6 (5.5)	16	73.7 (5.1)	8	72.8 (5.5)	11	71.8 (5.6)	14	70.6 (5.8)	19	72.0 (5.7)	13

^a^N denotes the sample size for each score on PRO-CTCAE item

**Table 6:** Item parameters of the non-PROMIS items after Stocking-Lord linking

NRS	*a*	*b1*	*b2*	*b3*	*b4*	*b5*	*b6*	*b7*	*b8*	*b9*	*b10*	*SL*^a^ *A*	*SL* *B*
Anxiety	2.99	− 0.63	− 0.08	0.33	0.63	0.85	1.13	1.41	1.73	2.16	2.79	0.96	−0.00
Depression	3.75	−0.23	0.20	0.51	0.80	1.01	1.30	1.47	1.82	2.22	2.61	0.90	−0.20
Fatigue	3.34	−0.97	−0.41	− 0.06	0.28	0.50	0.85	1.13	1.59	2.18	2.59	0.84	0.26
Pain intensity	3.26	−0.94	−0.20	0.32	0.75	1.02	1.40	1.77	2.16	2.71	3.15	1.59	−0.26
Sleep disturbance	2.66	−1.65	−0.98	−0.32	0.16	0.49	0.92	1.25	1.72	2.31	2.70	1.00	−0.05
**PRO-CTCAE**													
Anxiety severity	3.68	−0.38	0.65	1.59	2.47							0.96	−0.00
Anxiety frequency	3.95	−0.47	0.39	1.33	2.30							0.96	−0.00
Anxiety interference	3.43	0.34	1.09	1.83	2.56							0.96	−0.00
Depression: How often did you feel nothing could cheer you up?	4.07	0.06	0.81	1.58	2.22							0.90	−0.20
Depression: How often did you have sad/unhappy feelings?	3.96	− 0.74	0.33	1.26	2.28							0.90	−0.20
Depression: How much did feeling nothing could cheer you up interfere with activities?	4.11	0.34	1.08	1.73	2.39							0.90	−0.20
Depression: How much did sad/unhappy feelings interfere with activities?	3.90	0.15	0.94	1.62	2.32							0.90	−0.20
Depression: What was the severity of feelings that nothing could cheer you up at the worst?	4.61	0.20	0.89	1.71	2.40							0.90	−0.20
Depression: What was the severity of your sad/unhappy feelings at the worst?	3.80	−0.55	0.61	1.61	2.14							0.90	−0.20
Fatigue interference	4.95	−0.42	0.43	1.14	1.99							0.84	0.26
Fatigue severity	3.89	−0.85	0.25	1.24	2.06							0.84	0.26
Pain intensity	4.42	−1.02	0.36	1.43	2.47							1.58	−0.26
Pain interference	4.67	0.14	0.85	1.46	2.14							1.14	0.27
Sleep interference	2.90	−0.23	0.63	1.49	2.47							1.00	−0.05
Sleep severity	3.54	−0.58	0.25	1.28	2.23							1.00	−0.05

^a^SL A: Stocking-Lord multiplicative constant, SL B: Stocking-Lord additive constant. Stocking-Lord’s A and B constants are computed from the two sets of parameters for the common items so that their test characteristic curves become as similar as possible

Across domains, the score of zero on NRS was mapped to about 38.2 ± 3.3 on *T* scale, and the maximum score of ten on NRS to about 72.7 ± 2.2. In addition, the score of 50 which is the population norm on PROMIS scales was mapped to approximately 3 on NRS fatigue, 2 on pain intensity, 3 on NRS sleep disturbance, 2 on NRS anxiety, and 1 on NRS depression. The middle NRS categories tended to be close in terms of PROMIS scores (e.g., the NRS scores of 4, 5, and 6 in Table 3), which can be attributed to limited ability of categories of 4 and 6 to separate responders. Appendix D shows the item characteristic curves for the NRS items.

In terms of PRO-CTCAE, the (lowest/best) score of one corresponded to about 39.8 ± 3.6 on PROMIS *T* score metric depending on domains. The maximum score of five was mapped to about 72 ± 2 on PROMIS scale.

## Conclusions

Based on two different linking methods, we provide practical crosswalk tables that link PROMIS with 0–10 numeric rating scales (NRS) and PRO-CTCAE items in the following symptoms: pain, fatigue, anxiety, depression, and sleep. This is the first linking of these symptoms as measured by the PRO-CTCAE and NRS. Results based on both methods (fixed parameter and concurrent calibration with linking constants) were similar and consistent with each other. We tested whether the measures to be linked are highly similar in contents, highly correlated, and are likely to produce invariant linkages between subgroups. Through the IRT linking process, we found that a T score of 50 on PROMIS scale (the population norm) was aligned with NRS scores of 2 or 3 for domains other than depression, which was closer to 1. T scores of 50 were linked with level “2” responses on the PRO-CTCAE items across domains. Interestingly, the 11-level NRS items had only a slightly wider PROMIS score range compared to the 5-level PRO-CTCAE items. For example, NRS was equivalent to about 38 to 72 (span of 34) on average on PROMIS scale, while the 1–5 PRO-CTCAE score range was equivalent to PROMIS scores of about 41 to 72 (span of 31). Thus, the 5-level PRO-CTCAE scales and 11-level NRS scales map onto comparable score ranges on the common PROMIS scale.

## Discussion

The linking tables produced by this effort will have practical research and clinical value. One limitation is that we only used an IRT approach so cannot determine how our results compare to a non-IRT approach. Having this in mind, researchers can use the tables produced by the study to estimate group means on one measure even when the sample had been assessed with another. This has particular value for systematic review and meta-analysis of research questions raised around these important cancer symptoms. Clinically, it will be useful to compare cut scores for symptom severity, to help refine the actionability of patient response, and change in response, in treatment settings.

## Supplementary Information

**Additional file 1: Appendix A.** Standardized Mean Differences by Gender and Age (< 60 vs ≥ 60). Average Female subtracted by male scores, and average older (≥ 60) subtracted by younger (< 60) scores are presented. **Appendix B.** Comparison of Test Characteristic Curves and the Difference in Raw Score Values across the Scale between Concurrent Calibration with Linking Constants and Fixed Calibration. **Appendix C.** Item parameters from the fixed (x-axis) and SL-adjusted free calibrations (y-axis) with the identity lines. **Appendix D.** Item characteristic curves for NRS items.

## Data Availability

Data can be made available upon reasonable request to the principal investigator (J. Sloan). All requests will be reviewed.

## Abbreviations

PROM: Patient-reported outcome measure
HRQOL: Health-related quality of life
NRS: Numerical rating scale
PRO-CTCAE: PRO version of the Common Terminology Criteria for Adverse Events
PROMIS: PRO Measurement Information System
TCC: Test characteristic curve
SMD: Standardized mean difference
