# Correction to: Establishing a common metric for patient-reported outcomes in cancer patients: linking patient reported outcomes measurement information system (PROMIS), numerical rating scale, and patient-reported outcomes version of the common terminology criteria for adverse events (PRO-CTCAE)

**DOI:** 10.1186/s41687-020-00280-z

**Published:** 2021-02-05

**Authors:** Minji K. Lee, Benjamin D. Schalet, David Cella, Kathleen J. Yost, Amylou C. Dueck, Paul J. Novotny, Jeff A. Sloan

**Affiliations:** 1 Robert D. and Patricia E. Kern Center for the Science of Health Care Delivery, Mayo Clinic, 200 First St SW, Rochester, MN 55906 USA; 2 Department of Medical Social Sciences, Northwestern University Feinberg School of Medicine, 625 Michigan Ave, 27th Floor, Chical, IL 60611 USA; 3 Department of Health Sciences Research, Mayo Clinic, 200 First St SW, Rochester, MN 55905 USA; 4 Department of Health Sciences Research, Mayo Clinic, 13400 E. Shea Blvd, Scottsdale, AZ 85259 USA

**Correction to: J Patient Rep Outcomes 4, 106 (2020)**

**https://doi.org/10.1186/s41687-020-00271-0**

After publication of the original article [1], the authors notified some errors in Table 6 and in the Appendix 2:

Table 6 has errors in 2 parameters:

- For the cell that corresponds to “Pain Intensity (PRO-CTCAE)” and “SL A”, the correct number is **1.58** rather than 1.20;
- For the very next cell on the right that corresponds to “Pain Intensity (PRO-CTCAE)” and “SL B”, the correct number is **− 0.26** rather than − 1.04.

**Table 6:** Item parameters of the non-PROMIS items after Stocking-Lord linking

NRS	*a*	*b1*	*b2*	*b3*	*b4*	*b5*	*b6*	*b7*	*b8*	*b9*	*b10*	*SL*^*1*^ *A*	*SL* *B*
Anxiety	2.99	−0.63	−0.08	0.33	0.63	0.85	1.13	1.41	1.73	2.16	2.79	0.96	−0.00
Depression	3.75	−0.23	0.20	0.51	0.80	1.01	1.30	1.47	1.82	2.22	2.61	0.90	− 0.20
Fatigue	3.34	−0.97	−0.41	− 0.06	0.28	0.50	0.85	1.13	1.59	2.18	2.59	0.84	0.26
Pain intensity	3.26	−0.94	−0.20	0.32	0.75	1.02	1.40	1.77	2.16	2.71	3.15	1.59	−0.26
Sleep disturbance	2.66	−1.65	−0.98	−0.32	0.16	0.49	0.92	1.25	1.72	2.31	2.70	1.00	−0.05
PRO-CTCAE													
Anxiety severity	3.68	−0.38	0.65	1.59	2.47							0.96	−0.00
Anxiety frequency	3.95	−0.47	0.39	1.33	2.30							0.96	−0.00
Anxiety interference	3.43	0.34	1.09	1.83	2.56							0.96	−0.00
Depression: How often did you feel nothing could cheer you up?	4.07	0.06	0.81	1.58	2.22							0.90	−0.20
Depression: How often did you have sad/unhappy feelings?	3.96	−0.74	0.33	1.26	2.28							0.90	−0.20
Depression: How much did feeling nothing could cheer you up interfere with activities?	4.11	0.34	1.08	1.73	2.39							0.90	−0.20
Depression: How much did sad/unhappy feelings interfere with activities?	3.90	0.15	0.94	1.62	2.32							0.90	−0.20
Depression: What was the severity of feelings that nothing could cheer you up at the worst?	4.61	0.20	0.89	1.71	2.40							0.90	−0.20
Depression: What was the severity of your sad/unhappy feelings at the worst?	3.80	−0.55	0.61	1.61	2.14							0.90	−0.20
Fatigue interference	4.95	−0.42	0.43	1.14	1.99							0.84	0.26
Fatigue severity	3.89	−0.85	0.25	1.24	2.06							0.84	0.26
Pain intensity	4.42	−1.02	0.36	1.43	2.47							1.58	−0.26
Pain interference	4.67	0.14	0.85	1.46	2.14							1.14	0.27
Sleep interference	2.90	−0.23	0.63	1.49	2.47							1.00	−0.05
Sleep severity	3.54	−0.58	0.25	1.28	2.23							1.00	−0.05

^1^SL A: Stocking-Lord multiplicative constant, SL B: Stocking-Lord additive constant. Stocking-Lord’s A and B constants are computed from the two sets of parameters for the common items so that their test characteristic curves become as similar as possible

In Appendix 2, p. 12 shows old Pain Intensity item parameters; the correct parameters are below shown:

The newer one (correct one) shows

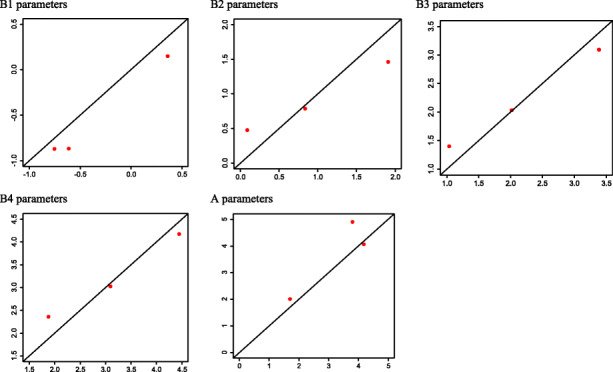



Furthermore the authors identified an error in the author name of Amylou C. Dueck. The incorrect author name is: Amy C. Dueck; The correct author name is: Amylou C. Dueck.

In the Results section, ‘’98.8%’’ should be replaced with ‘’71.2%’’ in the sentence “Breast cancer, lymphoma/myeloma, colorectal cancer, head/neck/gastroesophageal cancer, and lung cancer made up 98.8% of the patients”.

In the Linkage results section, ‘‘50-80’’ should be replaced with ‘‘60-80”,in the sentence “For all domains except pain intensity, the expected raw score values differed by less than |0.5| point across thetas. For pain intensity, in a higher T-score range of about 50–80, the difference in NRS score was larger than |0.5|’’.

The original article [1] has been corrected.
