# Correction to: Establishing a common metric for patient-reported outcomes in cancer patients: linking patient reported outcomes measurement information system (PROMIS), numerical rating scale, and patient-reported outcomes version of the common terminology criteria for adverse events (PRO-CTCAE)

**DOI:** 10.1186/s41687-023-00562-2

**Published:** 2023-07-10

**Authors:** Minji K. Lee, Benjamin D. Schalet, David Cella, Kathleen J. Yost, Amylou C. Dueck, Paul J. Novotny, Jeff A. Sloan

**Affiliations:** 1 Robert D. and Patricia E. Kern Center for the Science of Health Care Delivery, Mayo Clinic, 200 First St SW, Rochester, MN 55906 USA; 2 Department of Medical Social Sciences, Northwestern University Feinberg School of Medicine, 625 Michigan Ave, 27th Floor, Chical, IL 60611 USA; 3 Department of Health Sciences Research, Mayo Clinic, 200 First St SW, Rochester, MN 55905 USA; 4 Department of Health Sciences Research, Mayo Clinic, 13400 E. Shea Blvd, Scottsdale, AZ 85259 USA

## **Correction to: J Patient Rep Outcomes 4, 106 (2020).****https://doi.org/10.1186/s41687-020-00271-0**

The original publication of this article contained erroneous values in tables 3 and 4. The incorrect and correct values are shown in this correction article. The original article has been updated. This does not affect the conclusions of the article.

**Table:** 

Incorrect values in table 3			Correct values in table 3	
Sleep Disturbance^b^			Sleep Disturbance^b^	
T-Score (SE)	N		T-Score (SE)	N
34.9 (6.7)	174		35.0 (6.9)	174
41.0 (5.5)	228		41.0 (5.8)	228
45.6 (5.3)	316		45.5 (5.6)	316
49.5 (5.2)	276	→	49.4 (5.4)	276
52.3 (5.1)	193		52.3 (5.4)	193
55.0 (5.2)	224		55.0 (5.5)	224
57.6 (5.3)	127		57.6 (5.6)	127
60.3 (5.5)	122		60.3 (5.8)	122
63.6 (5.8)	76		63.6 (6.2)	76
66.8 (6.1)	23		66.7 (6.5)	23
70.5 (7.0)	20		70.1 (7.4)	20

**Table:** 

Incorrect values in Table 4			Correct values in Table 4	
Pain Intensity-Severity of pain at its worst			Pain Intensity-Severity of pain at its worst	
T-Score (SE)	N		T-Score (SE)	N
36.2 (5.7)	551		36.2 (5.7)	551
47.5 (5.0)	541	→	47.5 (5.0)	541
57.1 (4.5)	419		57.1 (4.5)	419
55.5 (4.6)	216		65.5 (4.6)	216
74.0 (5.2)	55		74.0 (5.2)	55

