# Supplementary material for: Establishing a common metric for patient-reported outcomes in cancer patients: linking patient reported outcomes measurement information system (PROMIS), numerical rating scale, and patient-reported outcomes version of the common terminology criteria for adverse events (PRO-CTCAE)
Source: J Patient Rep Outcomes. 2020 Dec 10;4:106. doi: 10.1186/s41687-020-00271-0 (PMC7728866; doi:10.1186/s41687-020-00271-0)
Supplement: Supplementary file 1 — Additional file 1: Appendix A. Standardized Mean Differences by Gender and Age (< 60 vs ≥ 60). Average Female subtracted by male scores, and average older (≥ 60) subtracted by younger (< 60) scores are presented. Appendix B. Comparison of Test Characteristic Curves and the Difference in Raw Score Values across the Scale between Concurrent Calibration with Linking Constants and Fixed Calibration. Appendix C. Item parameters from the fixed (x-axis) and SL-adjusted free calibrations (y-axis) with the identity lines. Appendix D. Item characteristic curves for NRS items. [file 41687_2020_271_MOESM1_ESM.docx]

Appendix A. Standardized mean differences by gender and age ( <60 vs ≥ 60). Average female subtracted by male scores, and average older (≥ 60) subtracted by younger (<60) scores are presented.

| Anxiety  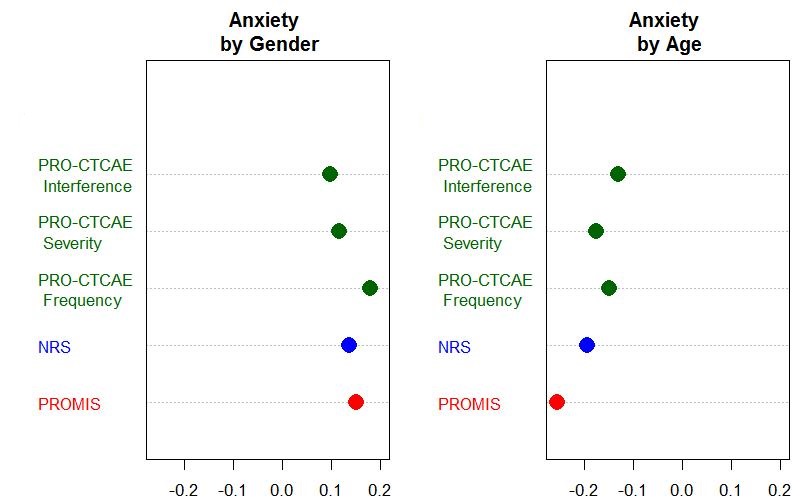 |
| --- |
| Depression  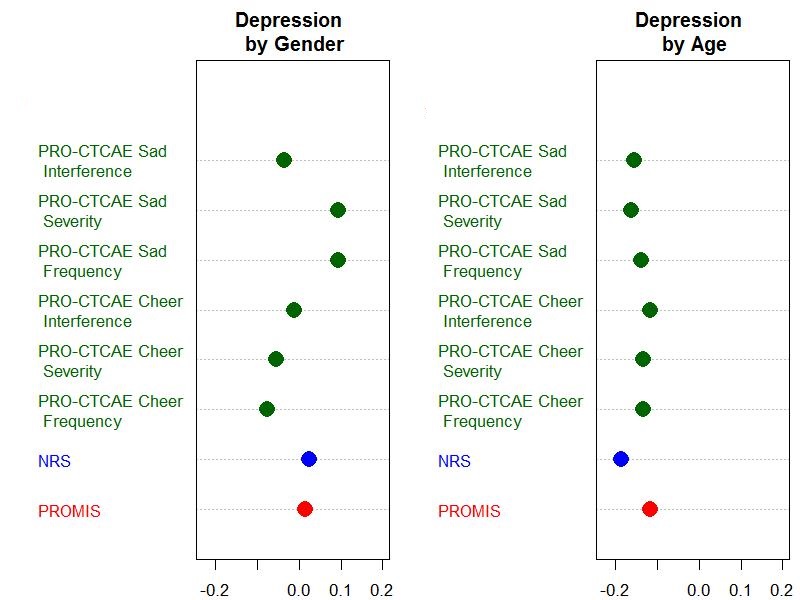 |

| Fatigue  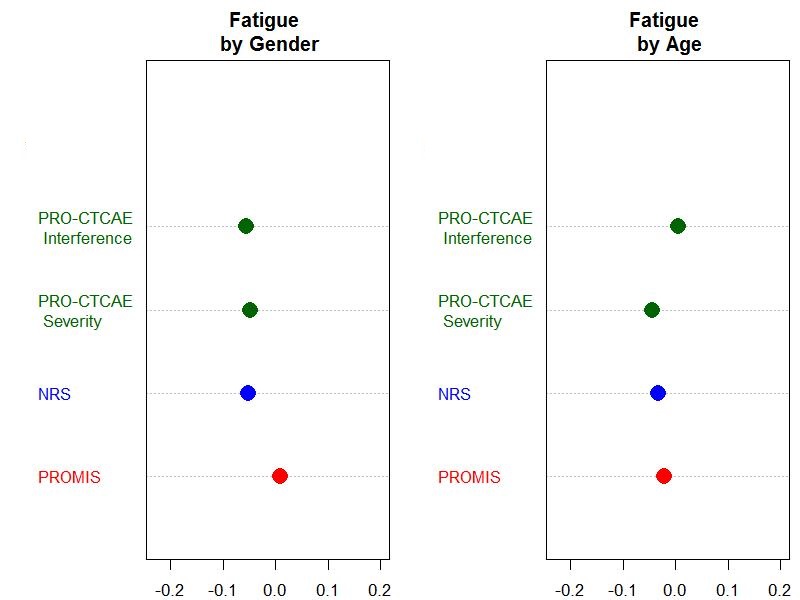 |
| --- |
| Pain Intensity  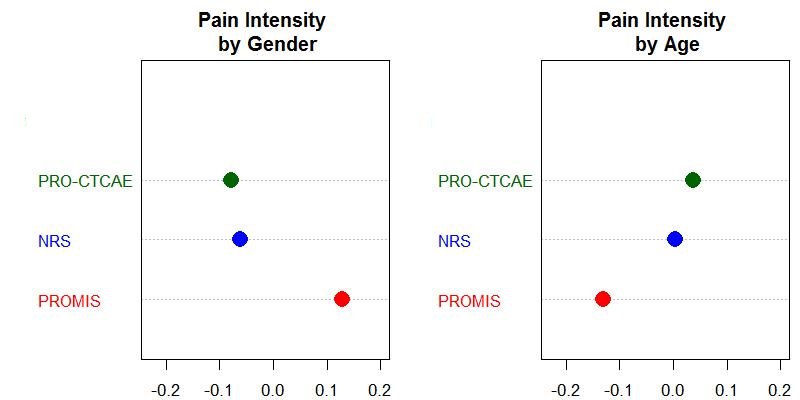 |

| Pain Interference  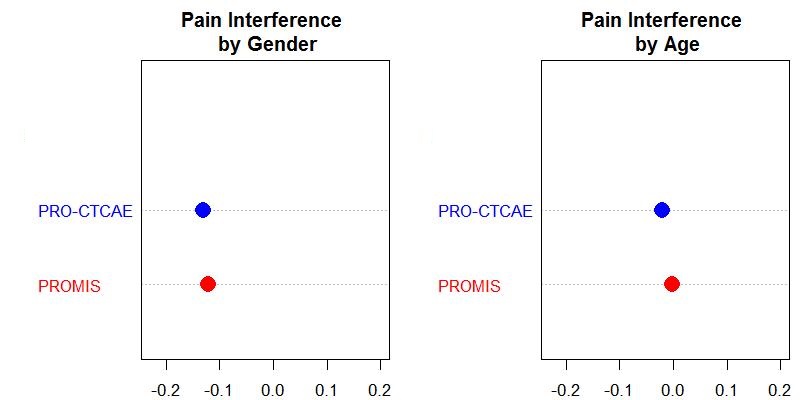 |
| --- |
| Sleep Disturbance  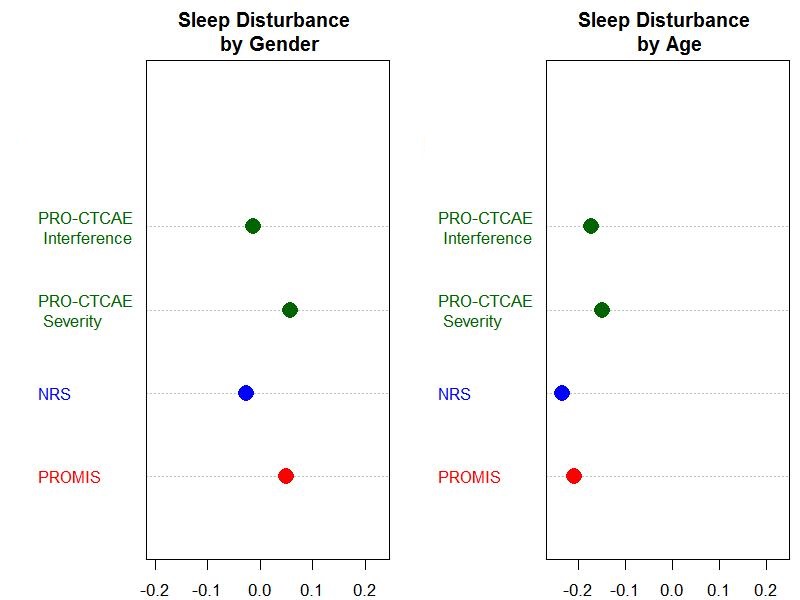 |

Appendix B. Comparison of test characteristic curves and the difference in raw score values across the scale between concurrent calibration with linking constants and fixed calibration

| Anxiety (NRS)  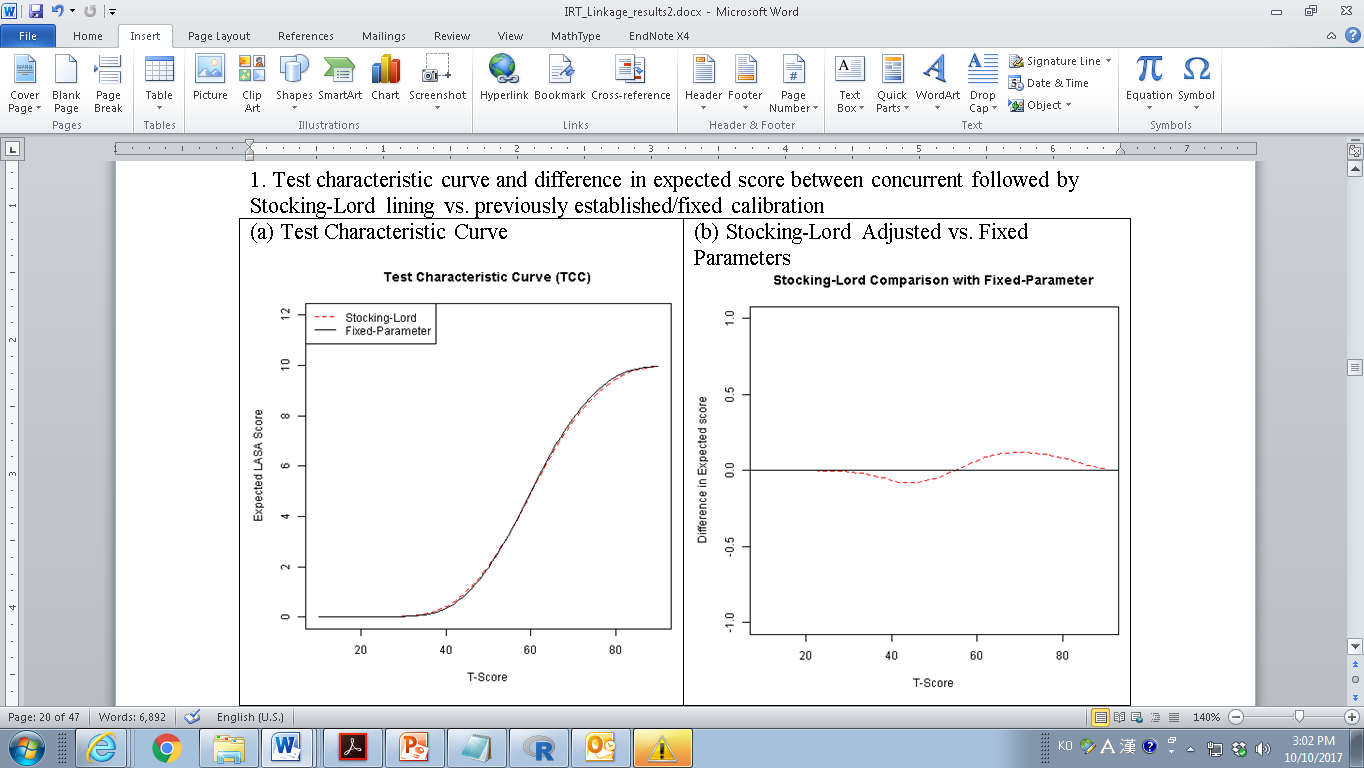 |
| --- |
| Anxiety (PRO-CTCAE – Severity)  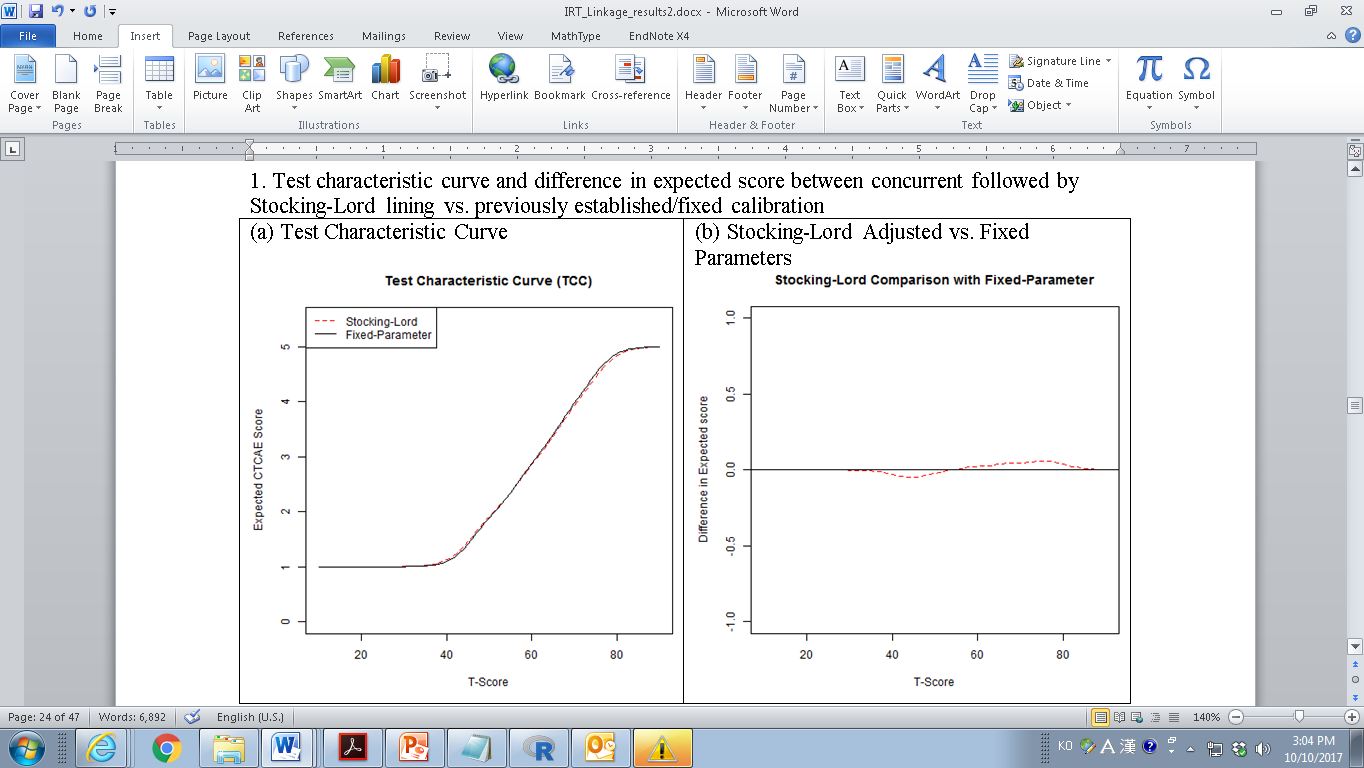 |

| Depression (NRS)  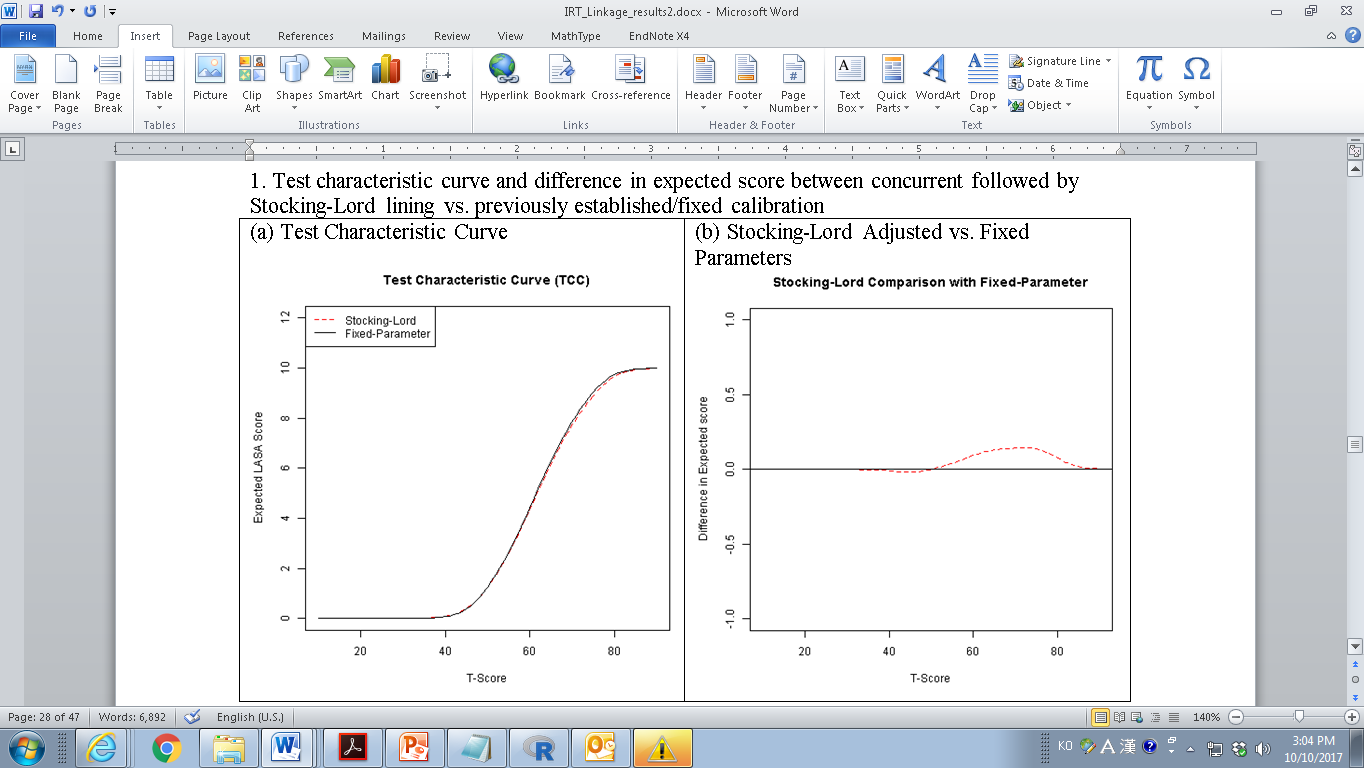 |
| --- |
| Depression (PRO-CTCAE severity of feeling nothing could cheer you up)  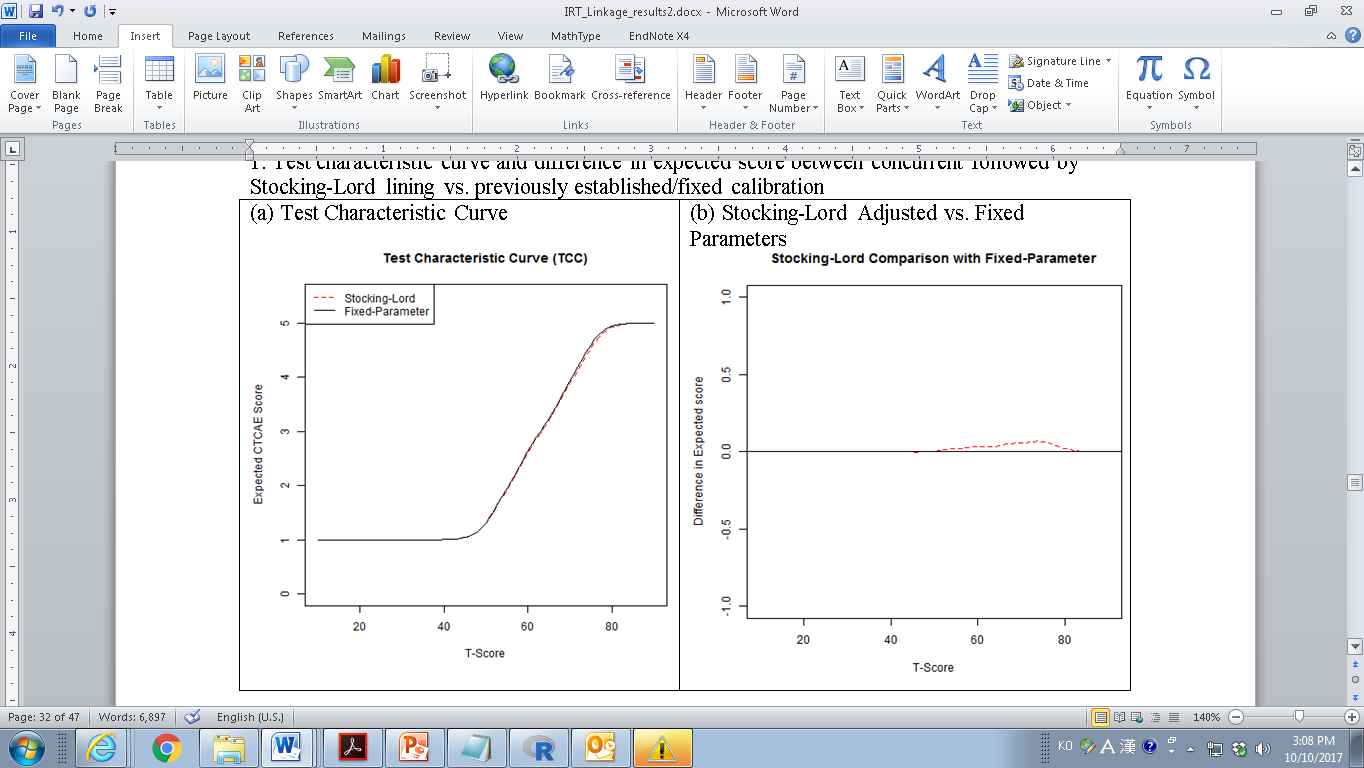 |

| Fatigue (NRS)  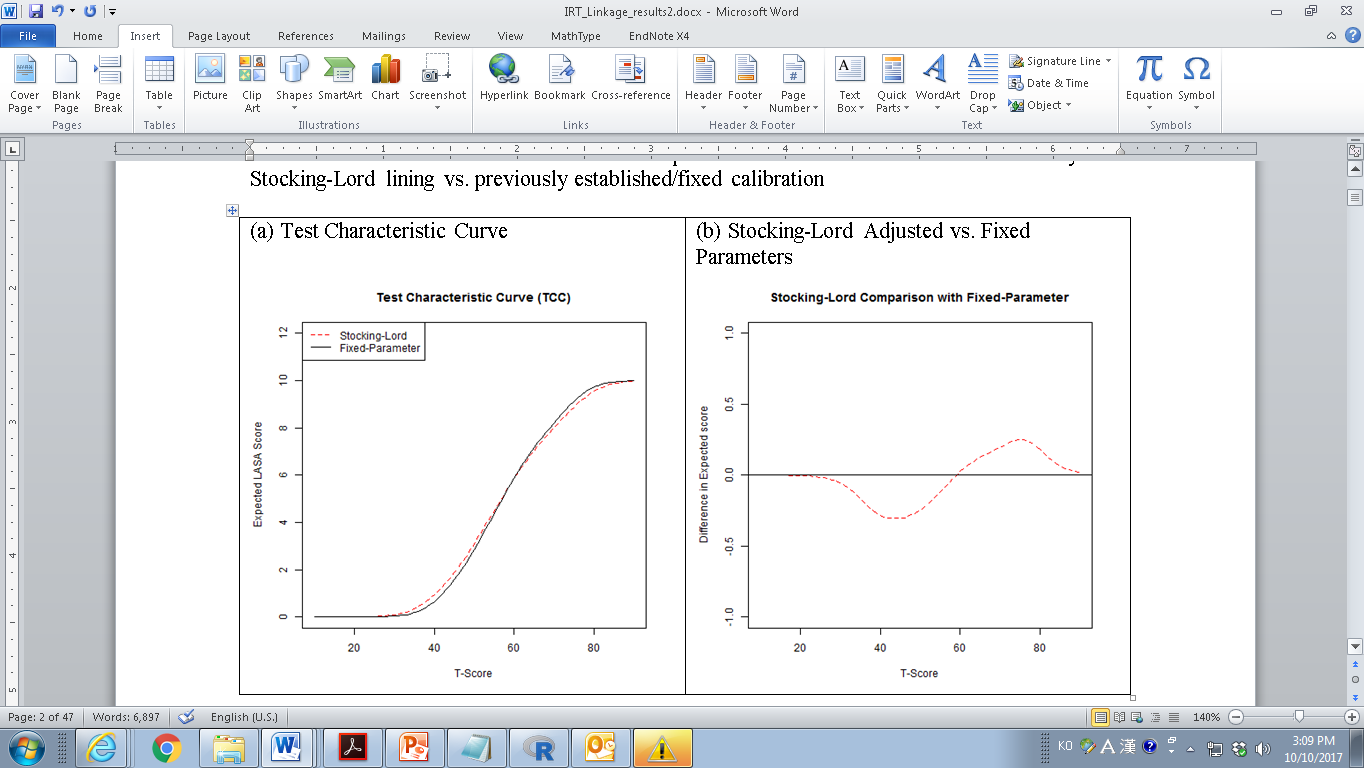 |
| --- |
| Fatigue (PRO-CTCAE severity)  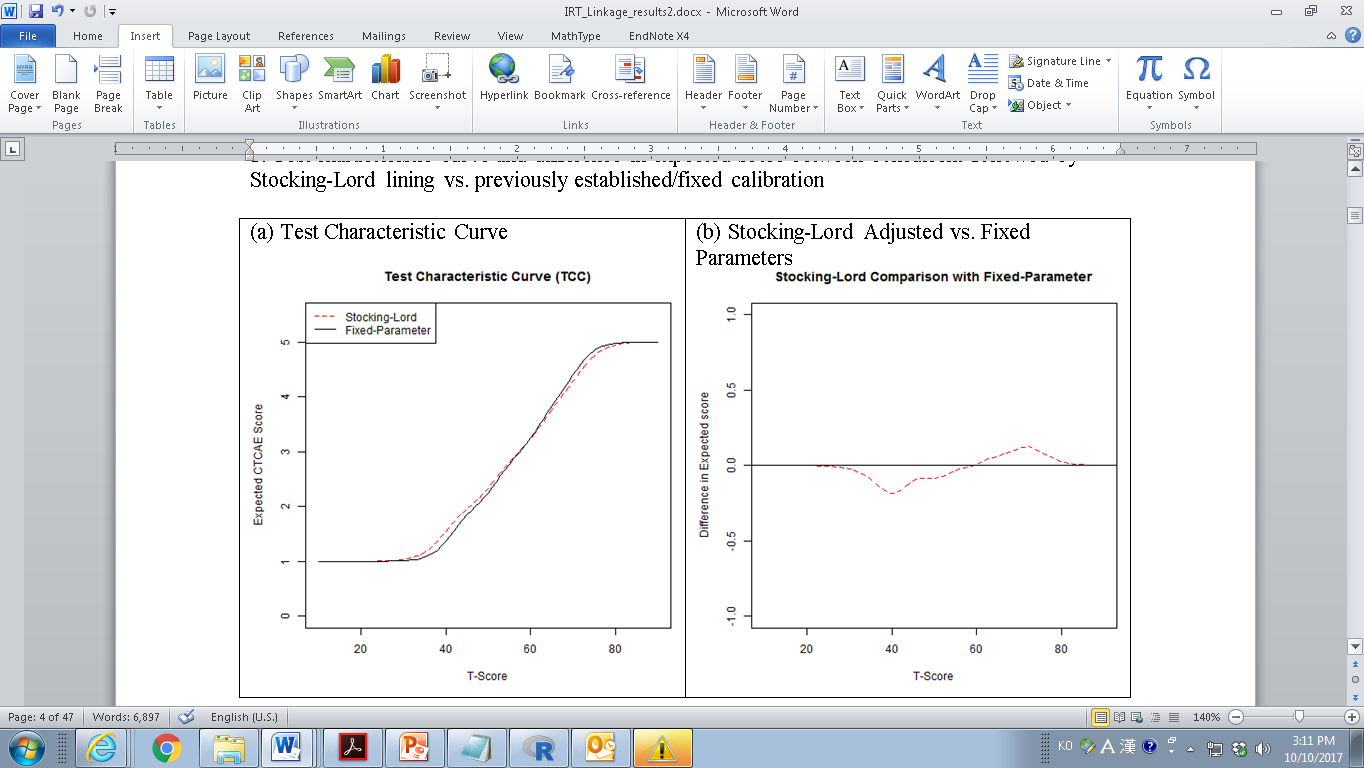 |

| Pain Intensity (NRS)  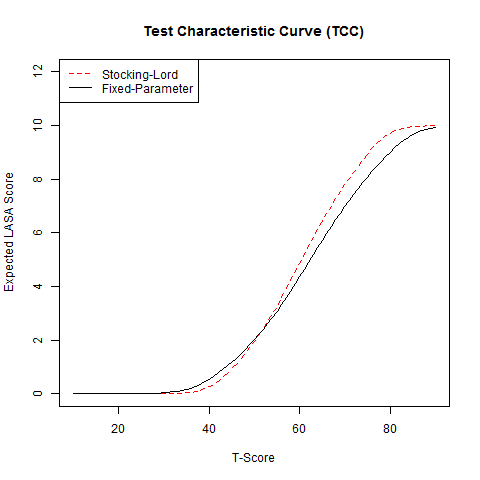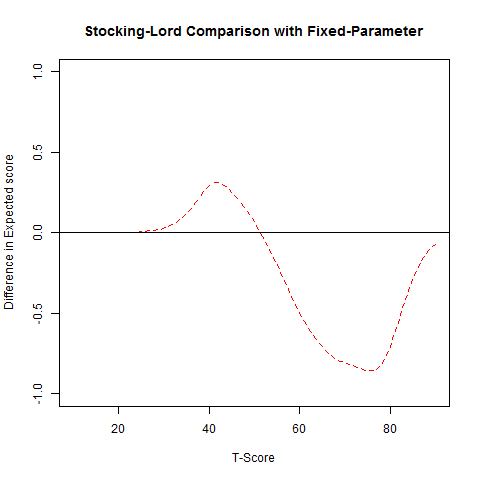 |
| --- |
| Pain Intensity (PRO-CTCAE severity)  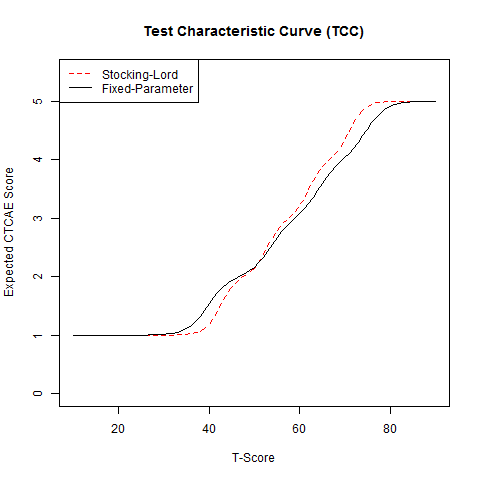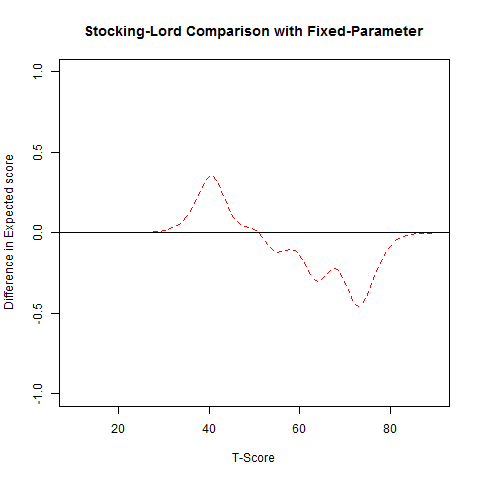 |

| Pain Interference (PRO-CTCAE interference)  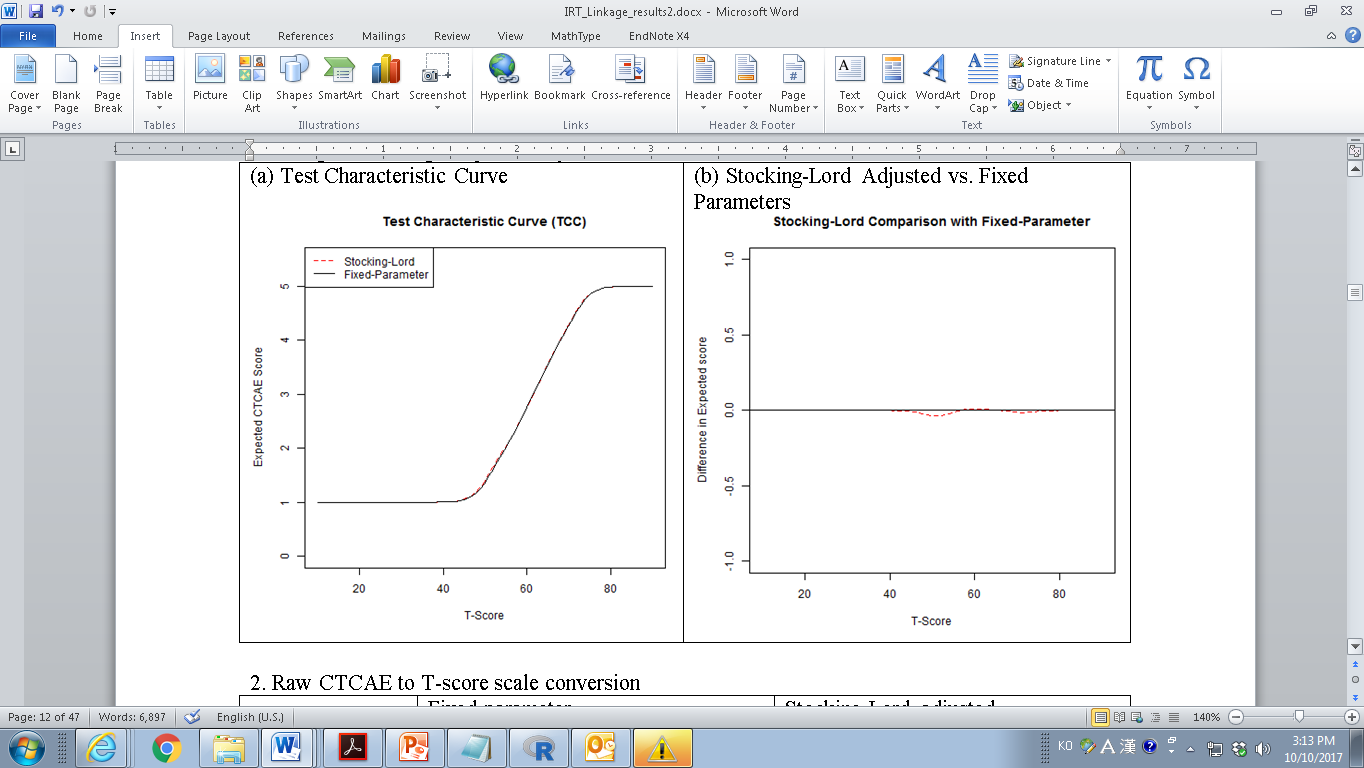 |
| --- |
| Sleep Disturbance (NRS – sleep quality reverse-coded)  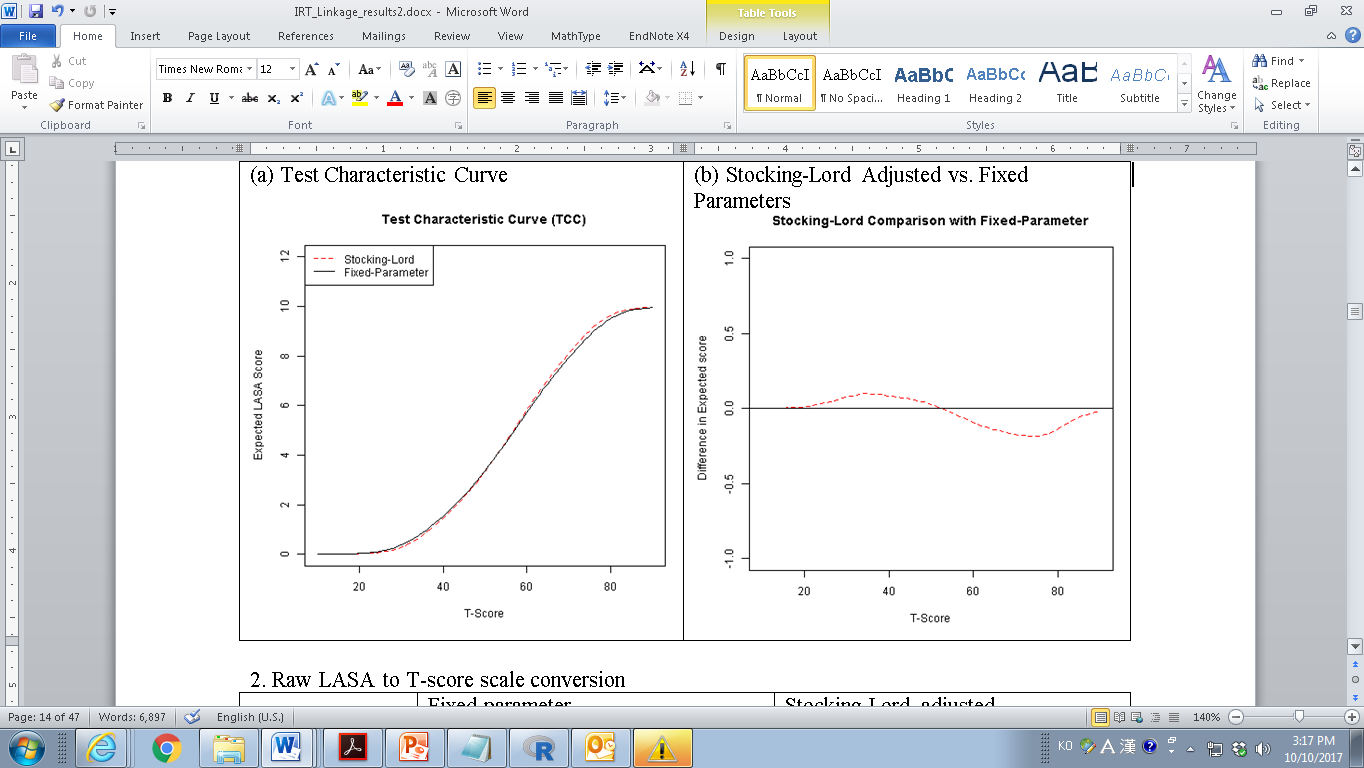 |

Sleep Disturbance (PRO-CTCAE severity)


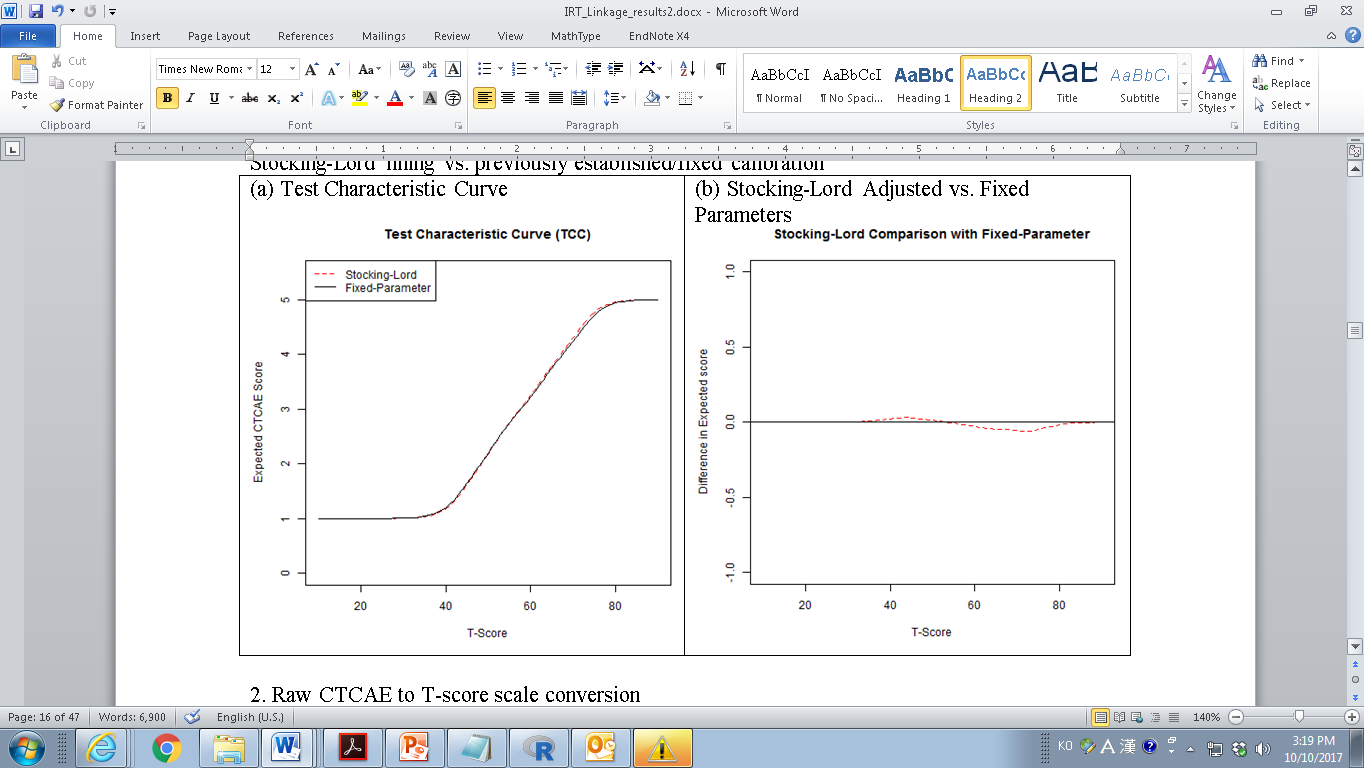


Appendix C. Item parameters from the fixed (x-axis) and SL-adjusted free calibrations (y-axis) with the identity lines

Anxiety (8 PROMIS anchor items)

| B1 parameters  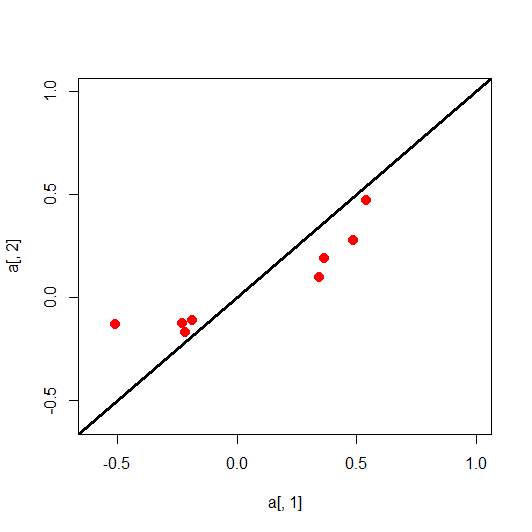 | B2 parameters  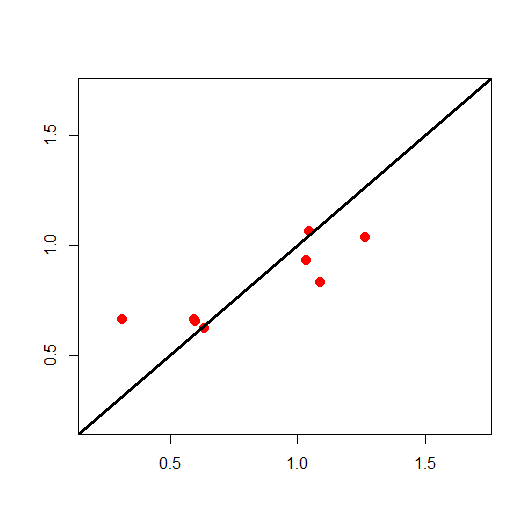 | B3 parameters  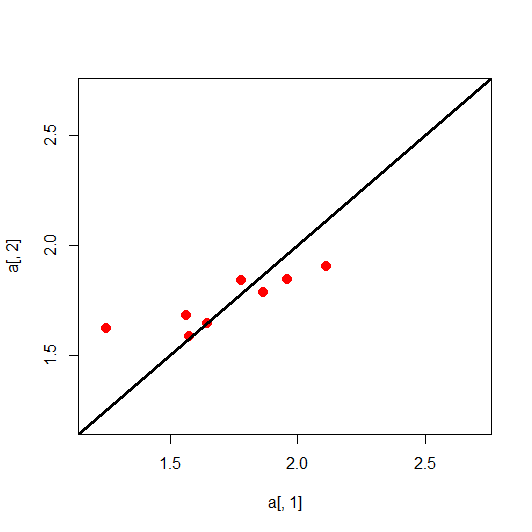 |
| --- | --- | --- |
| B4 parameters  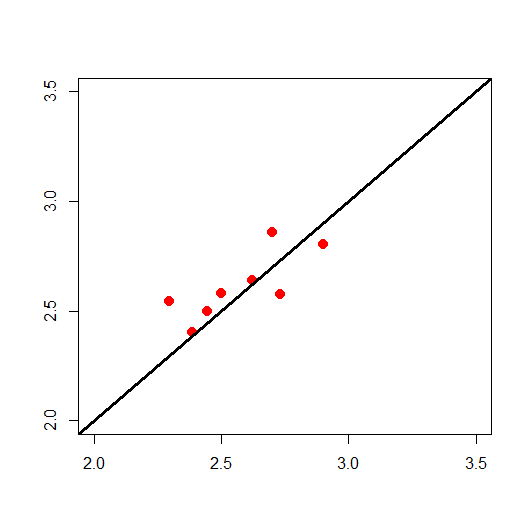 | A parameters  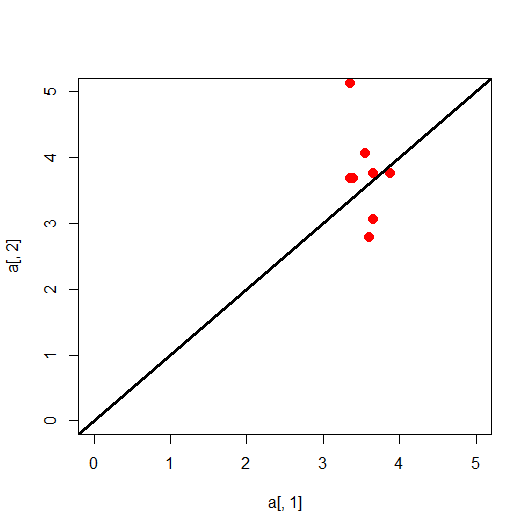 |  |

Note. B1-B4 denote the location parameters and A denotes the discrimination parameter.

Fatigue (9 PROMIS anchor items)

| B1 parameters  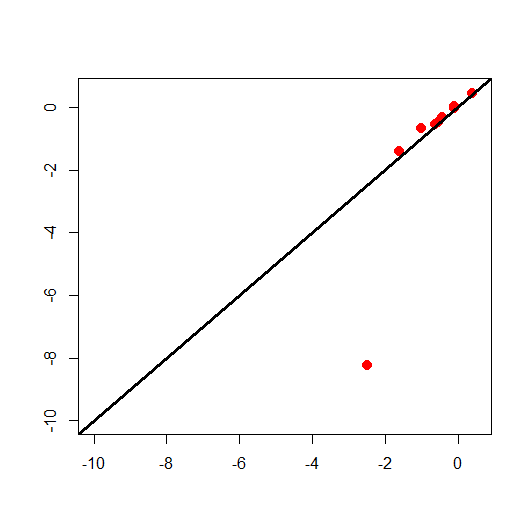 | B2 parameters  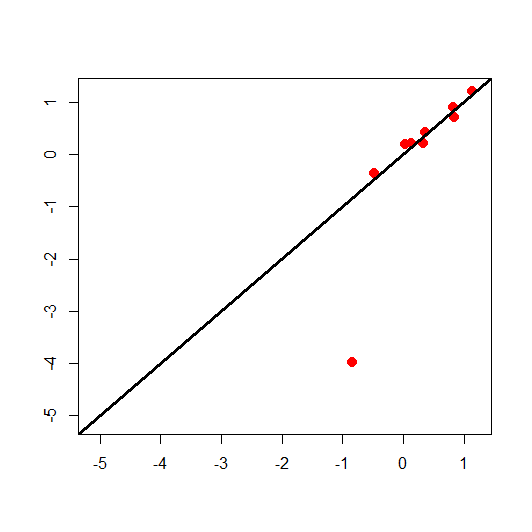 | B3 parameters  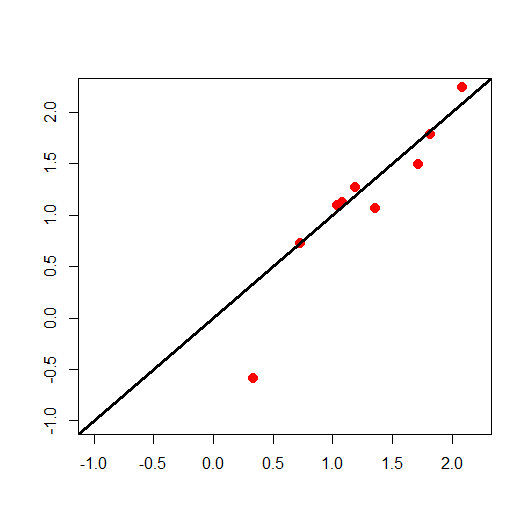 |
| --- | --- | --- |
| B4 parameters  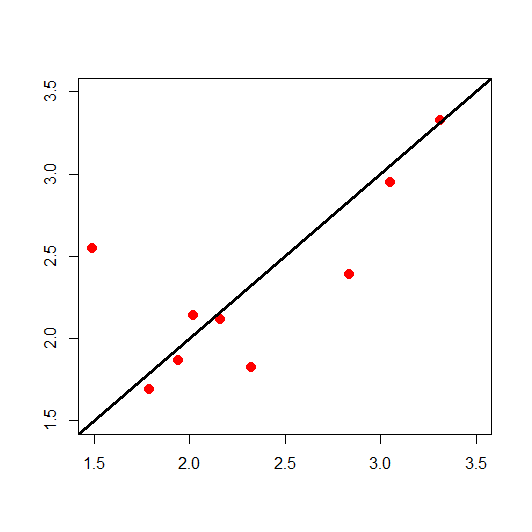 | A parameters  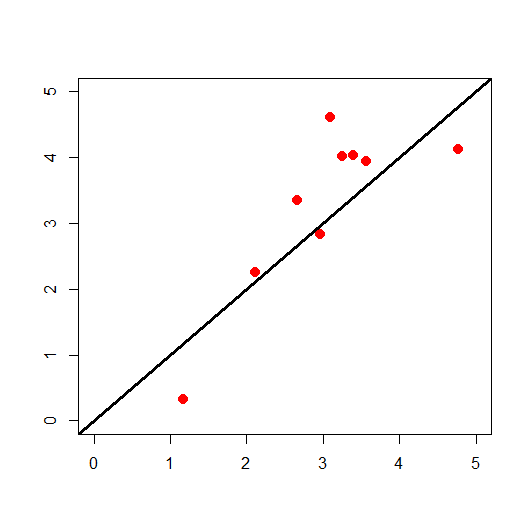 |  |

Note. B1-B4 denote the location parameters and A denotes the discrimination parameter.

Pain Intensity (3 PROMIS anchor items)

| B1 parameters   | B2 parameters   | B3 parameters   |
| --- | --- | --- |
| B4 parameters   | A parameters   |  |

Note. B1-B4 denote the location parameters and A denotes the discrimination parameter.

Sleep Disturbance (8 PROMIS anchor items)

| B1 parameters  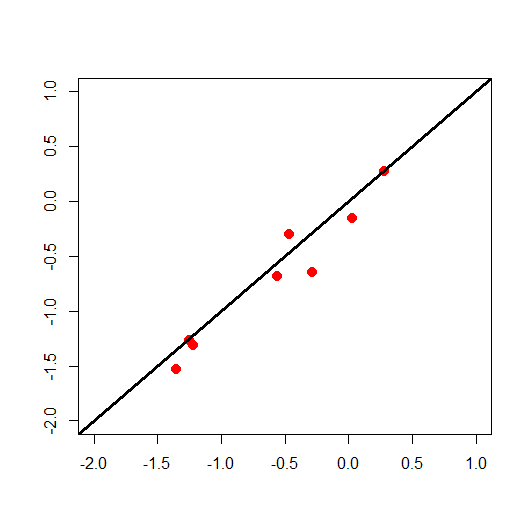 | B2 parameters  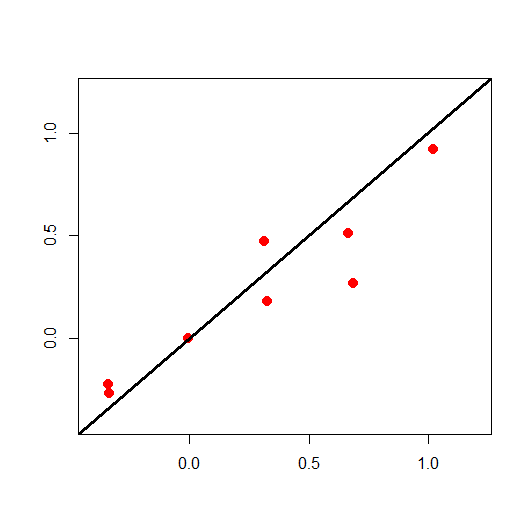 | B3 parameters  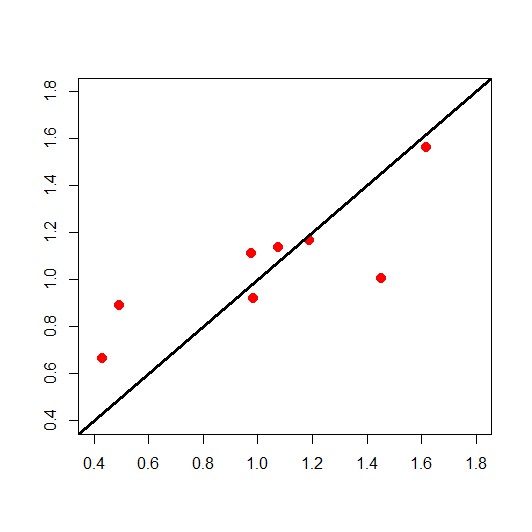 |
| --- | --- | --- |
| B4 parameters  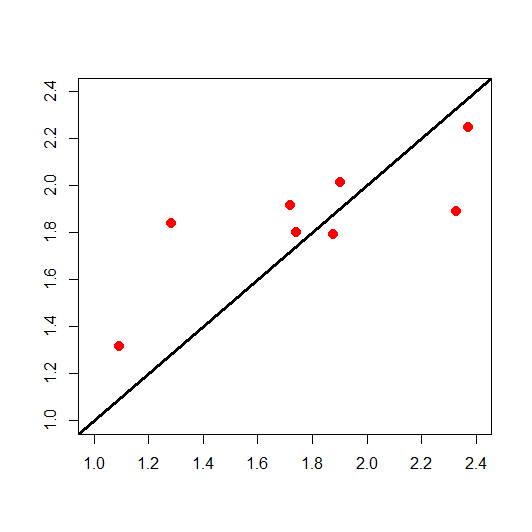 | A parameters  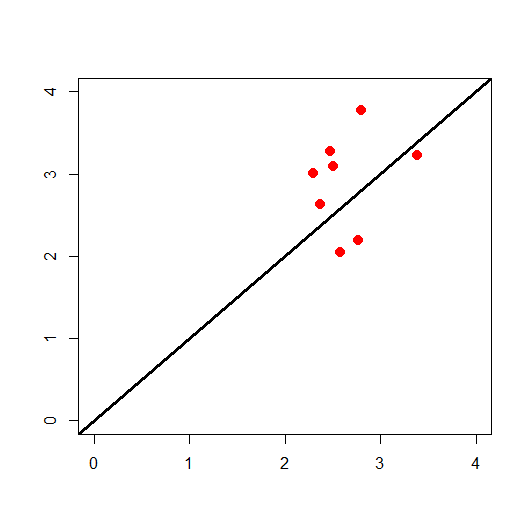 |  |

Note. B1-B4 denote the location parameters and A denotes the discrimination parameter.

Depression (8 PROMIS anchor items)

| B1 parameters  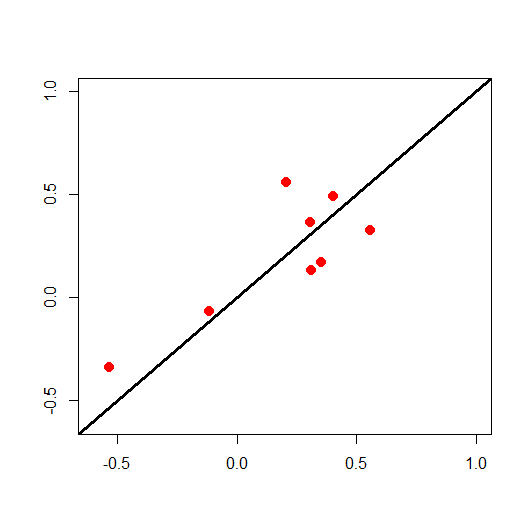 | B2 parameters  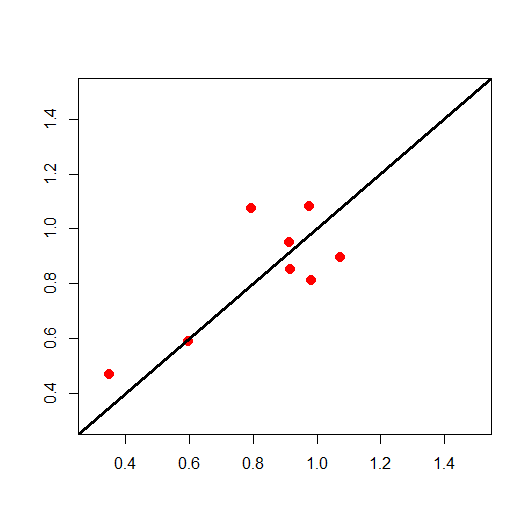 | B3 parameters  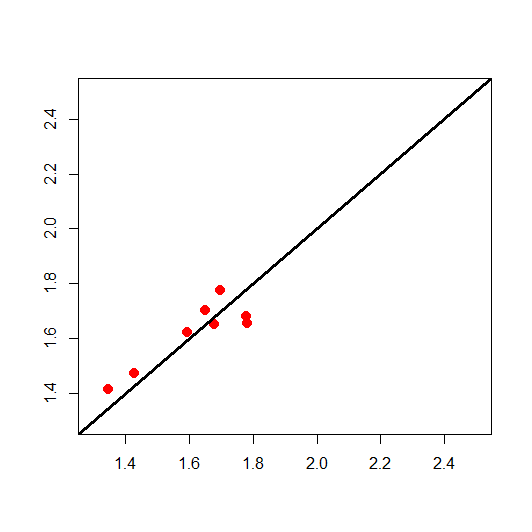 |
| --- | --- | --- |
| B4 parameters  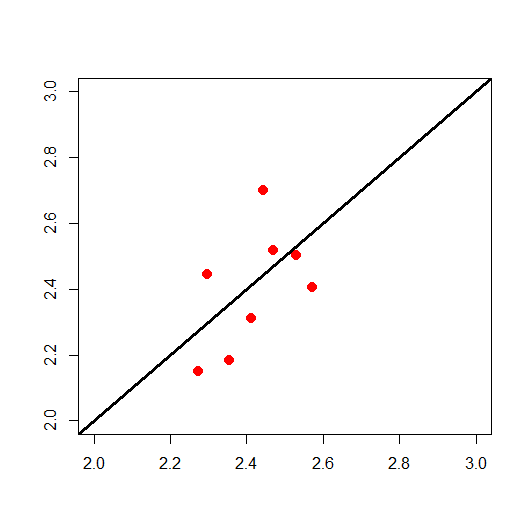 | A parameters  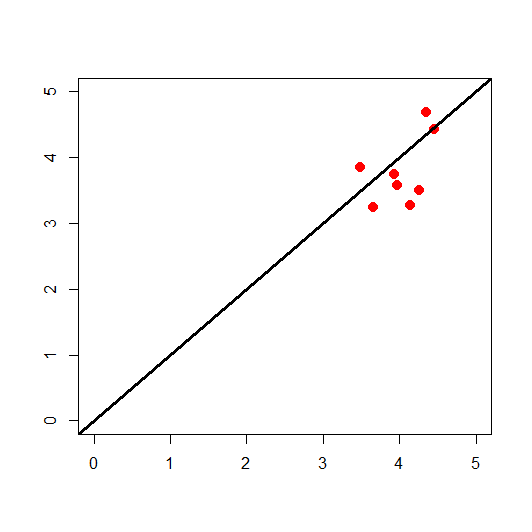 |  |

Note. B1-B4 denote the location parameters and A denotes the discrimination parameter.

Pain Interference (7 PROMIS anchor items)

| B1 parameters  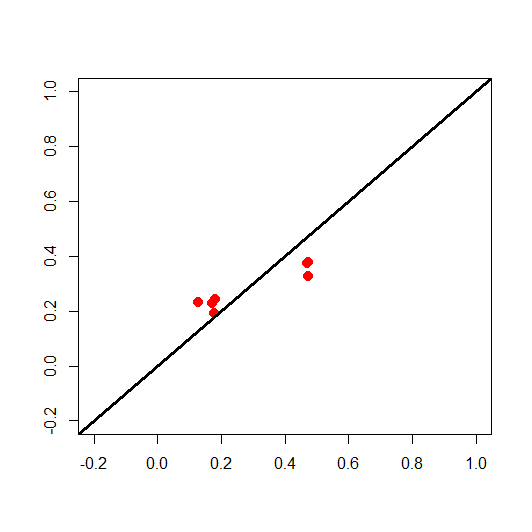 | B2 parameters  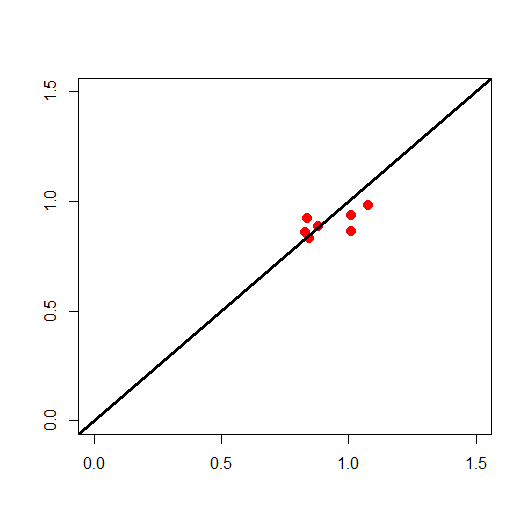 | B3 parameters  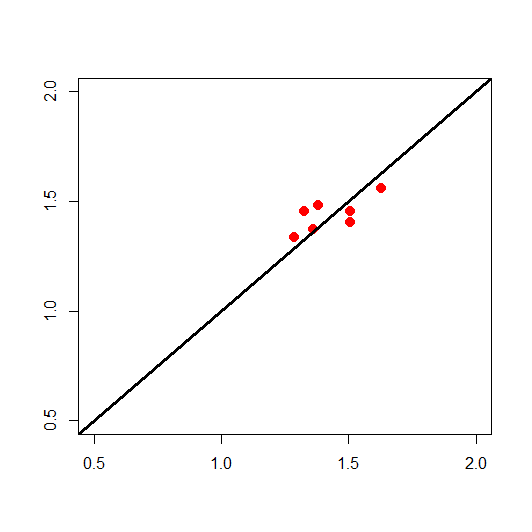 |
| --- | --- | --- |
| B4 parameters  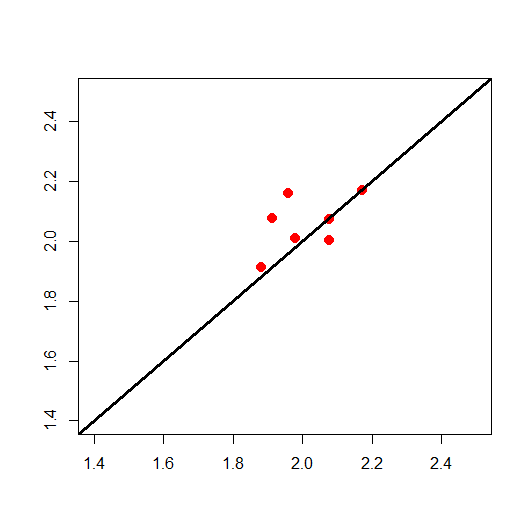 | A parameters  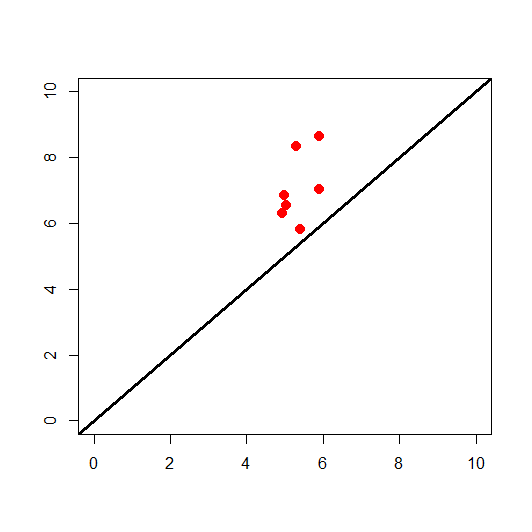 |  |

Note. B1-B4 denote the location parameters and A denotes the discrimination parameter.

Appendix D. Item characteristic curves for NRS items

| Anxiety fixed calibration   | Anxiety free calibration   |
| --- | --- |

| Fatigue fixed calibration   | Fatigue free calibration   |
| --- | --- |

| Depression fixed calibration   | Depression free calibration   |
| --- | --- |

| Pain intensity fixed calibration   | Pain intensity free calibration   |
| --- | --- |

| Sleep Disturbance fixed calibration (negatively worded)   | Sleep Disturbance free calibration (negatively worded)   |
| --- | --- |
